# DNMT2 inhibits anaplastic thyroid cancer progression by downregulating 5’tiRNA^Gly-GCC^ production

**DOI:** 10.1038/s41419-026-08488-5

**Published:** 2026-02-21

**Authors:** Ruixin Zhou, Baizhao Li, Mingyu Cao, Zhijing Wu, Fada Xia, Xinying Li

**Affiliations:** 1 Department of General Surgery, Xiangya Hospital of Central South University, Changsha, Hunan China; 2 National Clinical Research Center for Geriatric Disorders, Xiangya Hospital, Central South University, Changsha, Hunan Province China

**Keywords:** Thyroid cancer, Tumour-suppressor proteins

## Abstract

Complex tRNA methylation modifications collectively maintain the structural integrity and functional efficiency of tRNA. DNA methyltransferase 2 (DNMT2) regulates the m5C methylation status of tRNA, thereby reprogramming its structure and influencing cancer progression. However, the precise mechanisms through which DNMT2 affects tumor development via tRNA methylation remain insufficiently understood. In this study, we demonstrate that reduced DNMT2 expression promotes the progression of anaplastic thyroid carcinoma (ATC). Specifically, in ATC, DNMT2 catalyzes m5C38 methylation on three tRNAs: tRNA-Asp-GUC, tRNA-Gly-GCC, and tRNA-Val-AAC. Loss of DNMT2 leads to an increased abundance of 5’tiRNA^Gly-GCC^, generated by ANG-mediated cleavage of m5C38-hypomethylated tRNA-Gly-GCC. This 5’tiRNA^Gly-GCC^ directly binds to hnRNPH1, resulting in a reduction of its protein levels. Moreover, combined treatment with a 5’tiRNA^Gly-GCC^ inhibitor and doxorubicin hydrochloride significantly suppresses ATC progression in vivo. Thus, decreased DNMT2 expression facilitates ATC development by promoting the production of 5’tiRNA^Gly-GCC^. Our findings also highlight the considerable therapeutic potential of targeting 5’tiRNA^Gly-GCC^ in the treatment of ATC.

## Introduction

Currently, the global incidence of thyroid cancer (TC) continues to rise each year. TC is the most common endocrine tumor and ranks as the fifth most frequent cancer among women [1]. Despite anaplastic thyroid cancer (ATC) having the lowest incidence (comprising only 1%-3% of all TC), ATC exhibits the highest level of malignancy with a median survival of approximately 6 months [2]. Patients with ATC typically present with rapidly enlarging neck masses, and metastasis is often observed at the initial diagnosis [3]. This contributes to the limited efficacy of conventional treatments, such as surgery, radiotherapy, and chemotherapy. Currently, the combination of dabrafenib plus trametinib is considered the most effective treatment for ATC patients harboring BRAF V600E mutations [4], while effective systemic therapies remain lacking for other patient groups. Doxorubicin is a first-line drug for treating ATC, and doxorubicin monotherapy has been approved by the FDA for systemic chemotherapy in ATC patients [5, 6]. However, doxorubicin has limited efficacy in most patients and has failed to significantly improve long-term survival rates in ATC patients [6, 7]. Drug resistance poses a significant challenge to the treatment of ATC [8]. This underscores the urgent need to elucidate the molecular mechanisms driving ATC progression and to develop specific targeted therapies against key oncogenic drivers initiating ATC, with the ultimate goal of improving quality of life and overall survival rates for patients.

The methylation modification of 5-methylcytosine (m5C) constitutes a significant aspect of RNA post-transcriptional modification [9]. As an important component of protein translation, transfer RNA (tRNA) is also rich in m5C modifications. The enzymes NOL1/NOP2/SUN domain (NSUN) family 1-7 (NSUN1-7), particularly NSUN2, along with DNA methyltransferase 2 (DNMT2), catalyze m5C, thereby maintaining tRNA stability, ensuring translational fidelity, and regulating the production of tRNA-derived small RNAs (tsRNAs), including tRNA-derived fragment (tRF) and tRNA halves (tiRNA) [10]. Notably, DNMT2 is overexpressed in various cancers and is regarded as a potential therapeutic target [11]. However, our previous research has demonstrated that DNMT2 expression is downregulated in ATC compared to normal thyroid tissue [12]. This finding suggests that the prevailing studies on the oncogenic mechanisms of DNMT2 may not be applicable to ATC. Investigating the role of low DNMT2 expression in ATC could offer novel insights for the development of targeted therapeutics for this malignancy. Considering all the information, we hypothesize that in ATC cells, reduced DNMT2 expression enhances the creation of certain tsRNAs by decreasing the m5C levels in some tRNAs, thereby promoting ATC progression.

In this study, we discover that DNMT2 specifically methylates m5C38 of three tRNAs (tRNA-Asp-GUC, tRNA-Gly-GCC, and tRNA-Val-AAC) in ATC. Reduced expression of DNMT2 in ATC facilitates the production of 5’tiRNA^Gly-GCC^ by decreasing m5C levels, thereby advancing ATC progression. Concurrently, we observe that the combination of Antago-5’tiRNA^Gly-GCC^ and doxorubicin hydrochloride effectively impedes ATC progression. Therefore, our work reveals for the first time the oncogenic role of low-level DNMT2 in ATC and suggests a novel approach for the targeted treatment of ATC.

## Materials and Methods

### Cell culture

The human ATC cell lines 8305C (RRID: CVCL_1053), BHT101 (RRID: CVCL_1085), CAL62 (RRID: CVCL_1112), FRO (RRID: CVCL_6287), KHM-5M (RRID: CVCL_2975), and normal thyroid cell line Nthy-ori-3-1 (RRID: CVCL_2659) cells were purchased from Wuhan Pricella Biotechnology Co., Ltd. (Wuhan, China) in 2022 and authenticated by STR profiling. All cells were free of any contamination. All cells were cultured in medium containing 10% fetal bovine serum (Cat#A5256701, Gibco) and 1% penicillin/streptomycin at 37 °C and 5% CO_2_.

### Lentiviral transduction, Plasmid Transfection, and RNA interference

The knockdown and overexpression lentivirus of DNMT2, the plasmids of tRNA-Gly-GCC-WT, tRNA-Gly-GCC-C32A, and tRNA-Gly-GCC-C38A were all purchased from GENECHEM (Shanghai, China). When the cell density reached 50%, the cells were infected with lentivirus and selected with 10 μg/mL puromycin (Cat#HY-K1057, MedChemExpress, Shanghai, China) 48 h later. DNMT2-WT, DNMT2-C79A, DNMT2-mut, ANG-WT, ANG-mut, ANG-H37A, ANG-H138A, hnRNPH1-WT, hnRNPH1-RRM1, hnRNPH1-RRM2 and hnRNPH1-RRM3 were purchased from General Biol (Anhui) Co., Ltd (Anhui, China). According to the instructions, Lipofectamine 3000 (Cat#L3000015, Invitrogen, CA, USA) was used to transfect the plasmids and harvest the cells after 48 hours. ANG-targeting siRNA was purchased from RIBOBIO (Guangzhou, China), mimics and inhibitors of 5’tiRNA^Gly-GCC^ were purchased from ACCURATE BIOTECHNOLOGY(HUNAN) CO., LTD., ChangSha, China, and cells were transfected using AccuFect RNAi Transfection Kit (Cat#AG51018) purchased from ACCURATE BIOTECHNOLOGY (HUNAN) CO., LTD., ChangSha, China. The targets of shRNA and siRNA are shown in Supplementary Table 1.

### Animal studies

Animal experiments were conducted in the Center of Laboratory Animals of Xiangya Hospital, Central South University, and approved by the Animal Welfare and Ethics Committee of Xiangya Hospital, Central South University (No. XY20240228008). Female BALB/c nude mice aged 4 weeks were purchased from HUNAN SJA LABORATORY ANIMAL CO., LTD. Mice were randomly assigned to groups, with five mice (estimated based on previous experience) in each group. For the subcutaneous tumor model, 5×10^6^ NC-, SH-, or OE-DNMT2 KHM-5M cells and Matrigel (Cat#354248, Corning, USA) were mixed 1:1 and injected subcutaneously into randomized nude mice, and the length and width of the tumor were measured every four days. The tumor volume was calculated by the formula V = (length×width^2^)/2. The experiment was terminated after 32 days to collect the tumors of nude mice. The ATC lung metastasis model was established by tail vein injection of NC-, SH-, or OE-DNMT2 KHM-5M cells. Nude mice bearing KHM-5M xenograft tumors were injected with 4 mg/kg doxorubicin hydrochloride (Cat#25316-40-9, Selleck, TX, USA) or 10nmol Antago-5’tiRNA^Gly-GCC^ (RIBOBIO, Guangzhou, China) every three days. 0.9% saline was used as a control, and the tumor volume and nude mouse body weight were measured regularly. The experiment was terminated three weeks after drug injection. The relevant tests were conducted by researchers who were unaware of the group assignments.

### RT-qPCR

According to the manufacturer’s instructions, AG RNAex Pro Reagent (Cat#AG21101) purchased from ACCURATE BIOTECHNOLOGY(HUNAN) CO., LTD., ChangSha, China was used to extract total RNA from the cells. The Evo M-MLV RT Mix Kit with gDNA Clean for qPCR (Cat#AG11728, ACCURATE BIOTECHNOLOGY(HUNAN) CO., LTD, ChangSha, China) was used for reverse transcription of mRNA, while the miRNA 1st strand cDNA synthesis kit (Stem-loop) (Cat#AG11743, ACCURATE BIOTECHNOLOGY(HUNAN) CO., LTD, ChangSha, China) was utilized for reverse transcription of tRNA and tsRNA. Primers were purchased from Sangon Biotech (Shanghai, China) and ACCURATE BIOTECHNOLOGY (HUNAN) CO., LTD (Changsha, China), and sequences are described in Supplementary Table 2.

### Western blot

RIPA buffer (Cat#P0013B, Beyotime) with protease and phosphatase inhibitors (Cat#P002, NCM, Suzhou, China) was used to lyse cells and tumor specimens from nude mice. The total proteins were extracted after centrifuging for 15 min at 16,000 *g* and 4 °C, followed by denaturation for 10 min at 100 °C, then separated on a 10% SDS-PAGE gel and transferred to PVDF membranes (Cat#IPVH00010, MilliporeSigma, Darmstadt, Germany). After a 2 h block with 5% skimmed milk, the PVDF membranes were incubated overnight with the primary antibodies at 4 °C. Primary antibodies used in Western blotting included: Lamin B1 (Cat#12987-1-AP, Proteintech, Wuhan, China), GAPDH (Cat#60004-1-Ig, Proteintech, Wuhan, China), N-cadherin (Cat#GB111273, Servicebio, Wuhan, China), E-cadherin (Cat#GB11868, Servicebio, Wuhan, China), Vimentin (Cat#10366-1-AP, Proteintech, Wuhan, China), hnRNPH1 (Cat#14774-1-AP, Proteintech, Wuhan, China), DNMT2 (Cat#ab308120, Abcam, MA, USA) and ANG (Cat#18302-1-AP, Proteintech, Wuhan, China). The secondary antibodies were allowed to interact with the membrane for 120 min, at 25°C. The membranes were visualized using ECL Chemiluminescent Substrate Reagent Kit (Cat#P10300, NCM, Suzhou, China) and Amersham^TM^ ImageQuant 800 system.

### Immunohistochemistry (IHC) staining and H&E staining

IHC staining of tissue paraffin sections was performed using the IHC staining kit (Cat#SA1028, Boster, Wuhan, China). Primary antibodies used included: Ki-67 (Cat#27309-1-AP, Proteintech, Wuhan, China) and DNMT2 (Cat#19221-1-AP, Proteintech, Wuhan, China). Images were acquired using a PhenoImager HT (Akoya Biosciences, USA) and analyzed using Phenochart. H&E staining was performed using the H&E staining kit (Cat#AR1180, Boster, Wuhan, China), and images were obtained using a microscope (Leica, Germany).

### Immunofluorescence (IF) and Fluorescence in situ hybridization (FISH)

For immunofluorescence staining, briefly, the cell slides seeded with cells were fixed with 4% paraformaldehyde (Cat#G1101, Servicebio, Wuhan, China) for 10 min, followed by permeabilization of the nuclei with 0.25% Triton X-100 (Cat#G3068, Servicebio, Wuhan, China) for 10 min. After blocking with 3% BSA (Cat#GC305010, Servicebio, Wuhan, China) for 30 min, the primary antibodies were incubated at 4 °C overnight. The primary antibodies used were DNMT2 (Cat#19221-1-AP, Proteintech, Wuhan, China) and hnRNPH1 (Cat#14774-1-AP, Proteintech, Wuhan, China). Next, the fluorescent groups were linked using the DyLight 488-SABC Kit (Cat#SA1098, Boster, Wuhan, China). For FISH, after the above operations were performed until the nuclei were permeabilized, the 5’tiRNA^Gly-GCC^ was labeled with Cy3 using the Fluorescent In Situ Hybridization Kit (Cat#C10910, RIBOBIO, Guangzhou, China), and the probe sequence was GCATGGGTGGTTCAGTGGTAGAATTCTCGCC purchased from ACCURATE BIOTECHNOLOGY (HUNAN) CO., LTD. (Changsha, China). Images were captured using a fluorescence microscope (Leica, Germany) or a confocal microscope (Zeiss, Germany).

### Colony formation and transwell assay

For the colony formation assay, 800 cells were seeded into a six-well plate, and the medium was changed every three days. The assay was terminated when the number of cells in most individual colonies exceeded 50. The cells were fixed with 4% paraformaldehyde for 15 min and then stained with crystal violet stain for 20 min. For the Transwell experiment, briefly, 200 μL of serum-free culture medium containing 2 × 10^4^ cells was added to the upper chamber of the Transwell chamber (Cat#3422, Corning, USA). No Matrigel was added for the migration experiment, while 1 mg/mL Matrigel was added for the invasion experiment. 500 μL of culture medium containing 20% serum was added to the lower chamber. After 24 h of culture, the cells were fixed with 4% paraformaldehyde (Cat#G1101, Servicebio, Wuhan, China) for 15 min and stained with crystal violet for 20 min. The non-transferred cells in the chamber were gently wiped with a moistened cotton swab.

### Cell viability assay

To detect cell proliferation, 100 μl of culture medium containing 2.0 × 10^3^ cells was added to a 96-well plate and cultured in a cell culture incubator for 4 days. 10 μL Cell Counting Kit-8 (CCK8) (Cat#C6005, NCM, Suzhou, China) was added at a fixed time every day, and the absorbance was measured at 450 nm using a microplate reader after culturing at 37 °C for 30 min. For cytotoxicity assay, 100 μL of cell suspension containing 5 × 10^4^ cells was added to a 96-well plate, and after one day of culture at 37 °C, different concentrations of doxorubicin hydrochloride were used for 24 h. Then CCK8 solution was added as above. The results were processed with GraphPad Prism (v 10.3.1), and IC50 was calculated.

### RNA immunoprecipitation (RIP)

Use the RNA Immunoprecipitation (RIP) kit (Cat#Bes5101, BersinBio, Guangzhou, China) and follow the instructions. Briefly, remove gDNA after sufficient cell lysis. Incubate with the corresponding balanced magnetic beads after grouping and washing thoroughly. Extract RNA from each group with Trizol and detect the target RNA enrichment level by RT-qPCR.

### m5C Methylated RNA immunoprecipitation (MeRIP)

Enrich m5C-modified tRNA using the m5C MeRIP Kit (Cat#Bes5204-2, BersinBio, Guangzhou, China) according to the instructions. Briefly, collect enough cell pellets and extract total RNA with Trizol. Then use ultrasound or reagents to fragment tRNA and divide it into the IP group and the IgG group. Add m5C antibody to the IP group and IgG antibody to the IgG group. Add Protein A/G magnetic beads to different groups, incubate, and wash thoroughly. Extract the enriched m5C-modified total RNA and detect the target tRNA level by RT-qPCR.

### tRNA-BSseq

The tRNA bisulfite sequencing service was provided by CloudSeq Inc. (Shanghai, China). Total RNA was size-selected for the small RNA fraction (<200nt) with the MirVana Isolation Kit (ThermoFisher). The enriched small RNAs were demethylated with the AlkB enzyme mix for 100 min at 37 °C. Demethylated small RNA was bisulfite converted and purified using the EZ RNA methylation Kit (Zymo Research). Bisulfite-converted RNA was used for library construction with GenSeq® Small RNA Library Prep Kit (GenSeq, Inc.) by following the manufacturer’s instructions. All libraries were size-selected for tRNA fraction before sequencing and then sequenced on a NovaSeq platform (Illumina, Inc). Single-end reads were harvested from the Illumina sequencer and were quality controlled by Q30. Before data analysis, a tRNA library with 419 high-confidence tRNA sequences was adapted from the tRNAScan-SE library by appending CCA to tRNAs from the genomic tRNA database (http://gtrnadb.ucsc.edu/GtRNAdb2/genomes/eukaryota/Hsapi19/). After 3’ adaptor-trimming and low-quality reads removal by cutadapt software (v1.9.3), clean reads of BS-treated libraries were aligned to the tRNA library of the reference genome (UCSC hg19) by meRanGh (one component of meRanTK) software. The methylation status of each C within the genome was extracted by meRanCall (one component of meRankTK) software. meRanCompare (one component of meRanTK) software was used to identify differentially methylated sites (DMSs). DMSs on tRNA were annotated by home-made scripts.

### CHIRP-MS, RNA pulldown, and silver staining

Silver staining was performed using Fast Silver Stain Kit (Beyotime, Jiangsu, China) according to the instructions. CHIRP-MS (Aksomics, Shanghai, China) was used to search for proteins that specifically bind to 5’tiRNA^Gly-GCC^. Re-suspend cells with pre-cooling PBS buffer, crosslink with 3% formaldehyde at room temperature on an end-to-end shaker for 30 min. Quench crosslinking with 125 mM glycine for 5 min, spin at 1000RCF for 3 min, and discard supernatant, wash cell pellets twice with cooling PBS. For each 2 × ~107cells, add 1 mL Lysis buffer, sonicate the cell lysate in an ice-water bath, and check every 10 min until the cell lysate is no longer turbid. Spin at top speed, transfer supernatant to 2 volume of Hybridization Buffer, mix well, and incubate at 37 °C. Pre-bind probe (4 for TT, 1 for NC and PC, 100*pmol* per 2*×* 107 cells) to streptavidin beads for 30 min, wash out unbinding probe, and mix with cell lysate, hybridize at 37 °C overnight on an end-to-end shaker. Wash beads 5 times with 1 mL pre-warming Wash Buffer, 5 min per washing. At the last washing, transfer 1/20 beads for qPCR analysis. Add 100 µL Elution Buffer, 20U Benzonase, elute protein at 37 °C for 1 h. Transfer supernatant to a new low binding eppendorf tube. Wash beads with 100 µL Elution buffer once, and combine 2 supernatants. Reverse cross-linked sample at 95 °C, precipitate protein with 0.1% SDC and 10% TCA at 4 °C for 2 h. Spin at top speed, wash pellets with pre-cold 80% acetone 3 times. Next, the peptides were digested for LC-MS/MS. For each sample, ~ 1/2 peptides were separated and analyzed with a nano-UPLC (EASY-nLC1200) coupled to Q-Exactive mass spectrometry (Thermo Finnigan). Raw MS files were processed with MaxQuant (Version 1.5.6.0). RNA pulldown Kit (Bes5102, BersinBio) was used to verify the binding of 5’tiRNA^Gly-GCC^ to protein.

### LC-MS-based tRNA modification analysis

LC-MS-based tRNA modification analysis (Aksomics, Shanghai, China) was used to detect tRNA modification levels in cells. Total RNA samples are qualified by agarose gel electrophoresis and quantified using Nanodrop. tRNA was isolated from total RNA by the Urea-PAGE method. Then the tRNA was hydrolyzed to single dephosphorylated nucleosides by the enzyme mix. Pretreated nucleosides solution was deproteinized using a Satorius 10,000-Da MWCO spin filter. LC-MS analysis was performed on an Agilent 6460 QQQ mass spectrometer with an Agilent 1260 HPLC system using Multi-reaction monitoring (MRM) detection mode. LS-MS data were acquired using Agilent Qualitative Analysis software. MRM peaks of each modified nucleoside were extracted and normalized to the quantity of injected tRNA.

### In vitro tRNA cleavage assay

In vitro tRNA digestion was performed according to the previously described method [13]. Specifically, 100 ng/µL of tRNA was incubated in biochemically optimal buffer (50 mM MES pH 5.5, 10 mM NaCl, and 0.1 mg/mL BSA) at 90 °C for 2 min, followed by incubation at 25 °C for 3 min. MgCl_2_ was added to a final concentration of 20 mM and incubated at 37 °C for 5 min. After the final tRNA and 2.5 µM of recombinant ANG (Cat#HY-P7503, MedChemExpress) were incubated at 37 °C for the required time, proteinase K solution was added to the reaction mixture and incubated at 37 °C for 1 min. Immediately afterwards, an equal volume of 2×RNA Loading Dye (Cat#B0363S, NEW ENGLAND Biolabs) was added, and the tRNA digestion products were analyzed by 15% urea-denaturing polyacrylamide gel electrophoresis, followed by SYBR staining (Cat#S11494, Thermo Fisher Scientific). The tRNA was synthesized in vitro by General Biol (Anhui) Co., Ltd (Anhui, China) and labeled with Cy5 at the 5’ end.

### Patient samples

All paraffin sections of ATC patient tissue specimens were purchased from the Department of Pathology in Xiangya Hospital, Central South University. All the normal thyroid tissues were collected from Xiangya Hospital, Central South University, and approved by the Hospital’s Protection of Human Subjects Committee (No. 202004192) with informed consent from patients.

### Statistical analysis

All data from the three independent experiments that conform to a normal distribution are presented as mean ± standard deviation (SD). If not otherwise indicated, two-tailed Student’s *t*-test and one-way ANOVA were used to analyze the data. Survival curves were calculated using the Kaplan-Meier method and the log-rank test. Correlation analysis was conducted by the Spearman method. The statistical significance was set as *P* < 0.05.

## Results

### DNMT2 is down-expressed in ATC

**Fig. 1:**
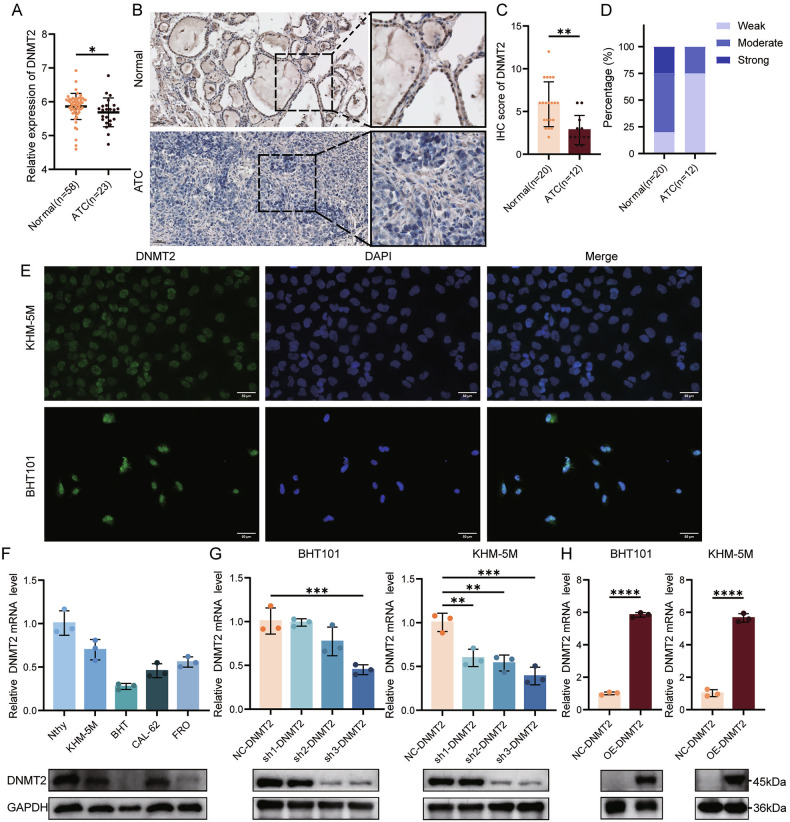
DNMT2 is under-expressed in anaplastic thyroid carcinoma (ATC). **A** Comparison of DNMT2 expression levels between normal thyroid tissue and ATC, as derived from datasets GSE33063 and GSE65144. **B**–**D** Immunohistochemistry (IHC) staining and quantitative analysis of DNMT2 expression in normal thyroid tissue (*n* = 20) versus ATC (*n* = 12). Scale bars, 50 μm. **E** Immunofluorescence analysis depicting the localization of DNMT2 in ATC cells, where green fluorescence indicates DNMT2 and blue fluorescence indicates the nucleus. Scale bars, 50 μm. **F** RT-qPCR and Western blot analyses demonstrate DNMT2 expression in normal thyroid cell lines compared to ATC cell lines. (*n* = 3) **G**, **H** RT-qPCR and Western blot evaluations of the transfection efficiency of sh-DNMT2 (*n* = 3, one-way ANOVA) and oe-DNMT2 (*n* = 3, Student’s *t*-test) in BHT101 and KHM-5M. All the data are shown as the mean ± SD. *P < 0.05; **P < 0.01; ***P < 0.001; ****P < 0.0001.

According to the level of DNMT2, we divided the TC samples from The Cancer Genome Atlas (TCGA) into two groups and performed Kaplan-Meier survival analysis. We found that high expression of DNMT2 was associated with longer progression-free survival (PFS), indicating that DNMT2 is a protective factor for TC prognosis (Supplementary Fig. 1A). Next, we processed the ATC data from GSE33630 [14] and GSE65144 [15] and the TC data from TCGA, then performed CIBERSORT immune infiltration analysis. The results showed that DNMT2 was negatively correlated with a variety of immunosuppressive cells, especially Treg cells (Supplementary Fig. 1B, C). At the same time, the results of CIBERSORT of TC data in TCGA showed that the CIBERSORT score of Tregs in TC was significantly higher than that in normal tissues (Supplementary Fig. 1D). To investigate the role of DNMT2 in ATC, we first integrated and analyzed 81 samples (58 NT, 23 ATC) from two datasets (GSE33630 and GSE65144). The results showed that DNMT2 was significantly down-regulated in ATC compared with normal thyroid tissue (Fig. 1A). To confirm this result, we performed immunohistochemistry (IHC) staining analysis on normal thyroid tissues and ATC tissues, and calculated the IHC score (Fig. 1B–D). The results showed that the expression level of DNMT2 in ATC was significantly downregulated. We performed immunofluorescence (IF) staining in ATC cell lines and found that DNMT2 was mainly localized in the cell nucleus (Fig. 1E). Next, we verified the low expression of DNMT2 in ATC cell lines and normal thyroid cell lines (Fig. 1F). BHT101 and KHM-5M cell lines were selected to construct stable cell lines overexpressing and knocking down DNMT2 by lentiviral transfection (Fig. 1G, H). In conclusion, our results indicate that DNMT2 is expressed at low levels and mainly localized in the nucleus in ATC cells.

### DNMT2 functions as an tumor suppressor in ATC

**Fig. 2:**
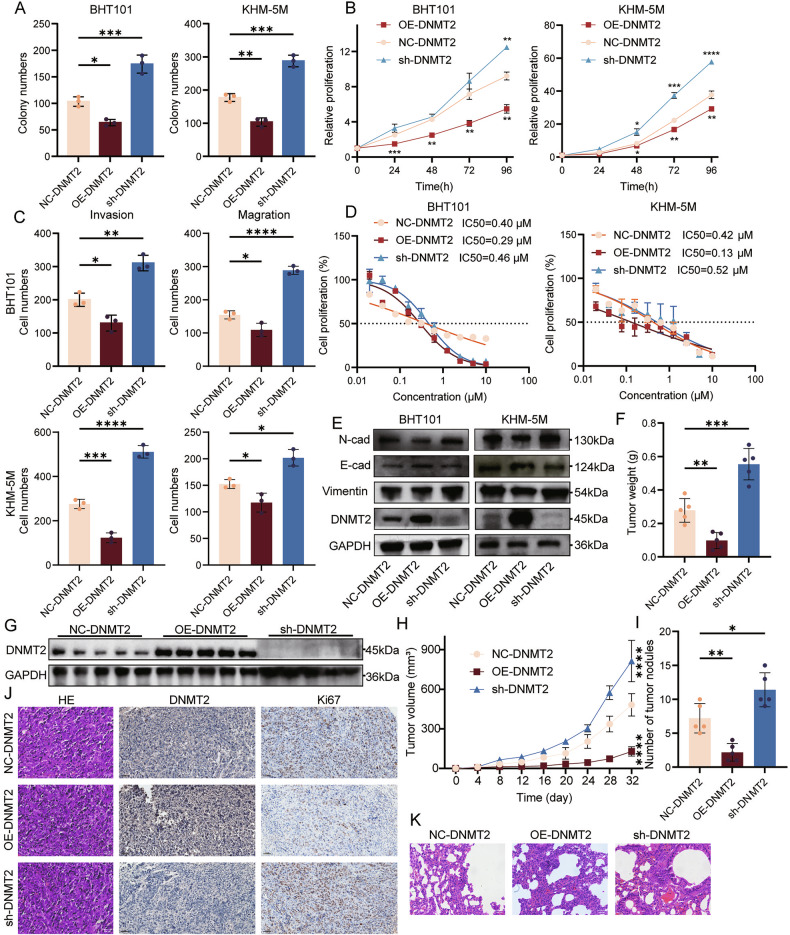
The low expression of DNMT2 promotes the progression of anaplastic thyroid carcinoma. **A** Colony-forming assays to evaluate the impacts of DNMT2 knockdown or overexpression in BHT101 and KHM-5M cells. (*n* = 3, one-way ANOVA). **B** Cell viability measured by CCK8 assay after DNMT2 knockdown or overexpression in BHT101 and KHM-5M cells with different times (0, 24, 48, 72, 96 h). (*n* = 3, two-way ANOVA). **C** Statistical analysis of transwell assays to show the invasion and migration abilities after DNMT2 knockdown or overexpression in BHT101 and KHM-5M cells. (*n* = 3, one-way ANOVA). **D** IC50 values of doxorubicin HCl in BHT101 and KHM-5M cells after DNMT2 knockdown or overexpression. **E** Western blot to analyze the EMT pathway related proteins expression levels in BHT101 (right) and KHM-5M (left) cells. (*n* = 3). **F** Tumor weights comparison in each group. (*n* = 5, one-way ANOVA). **G** Western blot to analyze the DNMT2 levels in each group. (*n* = 5). **H** Tumor growth curves of each group. (*n* = 5, two-way ANOVA). **I** Number of metastatic nodules on the lungs in each group. (*n* = 5, one-way ANOVA). **J** Representative images from H&E staining (Scale bars, 100 μm), DNMT2 and Ki-67 IHC staining (Scale bars, 50 μm) for tumor tissue in each group. **K** Representative images from H&E staining of the lungs of metastatic model mice. Scale bars, 100 μm. All the data are shown as the mean ± SD. *P < 0.05; **P < 0.01; ***P < 0.001; ****P < 0.0001.

Since ATC grows rapidly and invades and metastasizes highly, we explored the effect of reduced DNMT2 expression on ATC phenotype in vitro and in vivo. Cloning assays (Fig. 2A; Supplementary Fig. 2A) and CCK8 assays (Fig. 2B) demonstrated that low DNMT2 expression promotes ATC cell proliferation, while high DNMT2 expression suppresses it. Transwell experiments further showed that decreased DNMT2 expression enhances the invasive and metastatic capabilities of ATC cells (Fig. 2C; Supplementary Fig. 2B). At the same time, DNMT2 altered the sensitivity of ATC cells to doxorubicin hydrochloride. Knocking down DNMT2 increased the IC50 of ATC cells for doxorubicin hydrochloride, while overexpressing DNMT2 had the opposite effect (Fig. 2D). Western blot analysis of the epithelial-mesenchymal transition (EMT) pathway proteins found that the EMT pathway was activated after DNMT2 knockdown, which was inhibited after overexpressing DNMT2 (Fig. 2E).

Next, we further verified the effect of DNMT2 on ATC cells in nude mice. Compared with the NC-DNMT2 group, nude mice subcutaneously bearing with sh-DNMT2 cells exhibited faster tumor growth (Fig. 2G; Supplementary Fig. 2C), as reflected by increased tumor volume (Fig. 2H) and greater tumor weight (Fig. 2F). Overexpression of DNMT2 inhibited tumor growth in nude mice. H&E and IHC staining of the tumor tissues revealed that Ki-67 was highly expressed in the sh-DNMT2 group but lowly in the oe-DNMT2 group (Fig. 2J), which further indicated the tumor-suppressor role of DNMT2. The experimental lung metastasis model was used to evaluate the effect of DNMT2 on ATC metastasis to the lung (Supplementary Fig. 2D). Pulmonary metastatic nodule counts (Fig. 2I) and H&E staining (Fig. 2K) confirmed that overexpression of DNMT2 suppressed lung metastasis of ATC.

### DNMT2 regulates ATC progression by modulating m5C modification of specific tRNAs

**Fig. 3:**
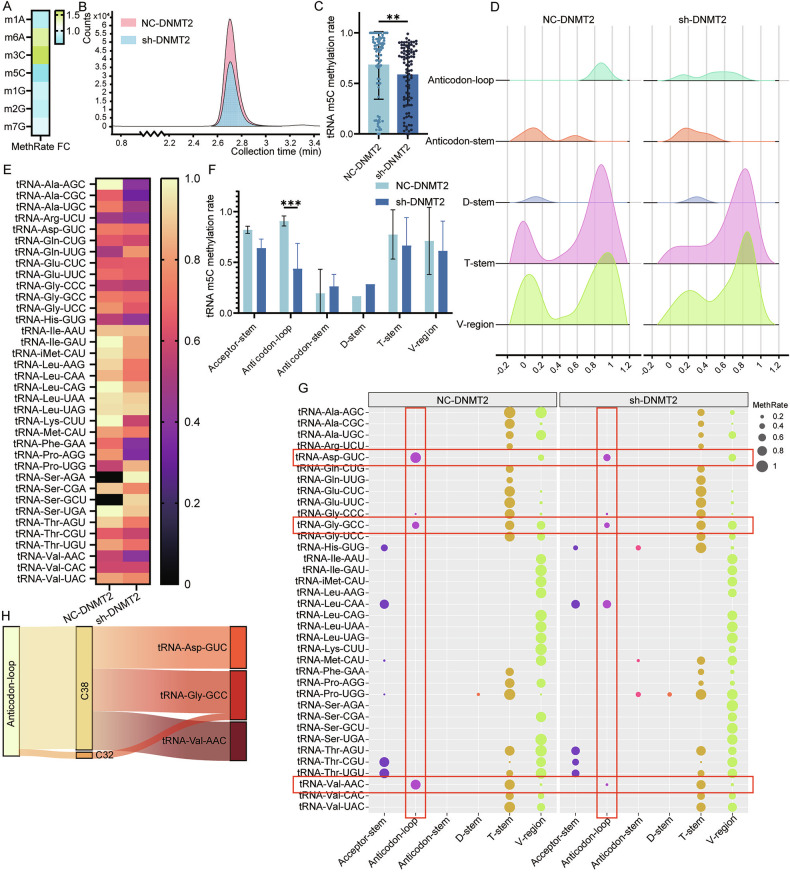
DNMT2 participates in the m5C methylation modification of tRNA C38. **A** Heat map of methylation rate fold change between NC-DNMT2 and sh-DNMT2. Green part of the heat map indicates upregulated methylation rate, blue part of the heat map indicates downregulated methylation rate. **B** LC-MS shows the change of m5C level between NC-DNMT2 group and sh-DNMT2 group. **C** Bar graph reflecting all tRNA m5C methylation rate in NC-DNMT2 group and sh-DNMT2 group. (Student’s *t*-test). **D** Ridgeline plot comparing per tRNA secondary structure m5C methylation rate in NC-DNMT2 group and sh-DNMT2 group. **E** Heat map of each tRNA m5C methylation rate in two groups. **F** Bar graph analyzing per tRNA secondary structure m5C methylation rate in two groups. (Two-way ANOVA). **G** Bubble plot showing m5C methylation rate (bubble size) of each secondary structure of each tRNA in NC-DNMT2 group (left) and sh-DNMT2 (right). **H** Sankey diagram showing m5C site in anticodon-loop of tRNA. All the data are shown as the mean ± SD. **P < 0.01; ***P < 0.001.

We used LC-MS to analyze tRNA methylation modification levels in NC-DNMT2 cells and sh-DNMT2 cells. Among all detected methylation types, m5C levels decreased significantly following DNMT2 knockdown (Fig. 3A, B). We then analyzed the m5C levels of tRNA in ATC cells by RNA-BSseq. The results showed that the overall m5C methylation rate of tRNA decreased significantly after knocking down DNMT2 (Fig. 3C, E). The secondary structure of tRNA includes acceptor-stem, anticodon-loop, anticodon-stem, D-loop, D-stem, T-loop, T-stem and V-region. Analysis of the m5C methylation rates occurring in these secondary structures revealed that the reduction in m5C levels caused by knockdown of DNMT2 only occurred in the anticodon loop (Fig. 3D, F). Next, we analyzed the methylation rates of the secondary structures of each tRNA one by one and found that only the m5C methylation rate of tRNA-Asp-GUC, tRNA-Gly-GCC and tRNA-Val-AAC decreased (Fig. 3G). These findings indicate that in ATC cells, DNMT2 mainly methylates the anticodon loops of these three tRNAs. At the same time, we also observed that m5C modification occurred primarily at the C38 site, with a smaller proportion detected at the C32 site of tRNA-Gly-GCC (Fig. 3H).

### Exploration of the molecular mechanism of DNMT2-specific modification of tRNA

**Fig. 4:**
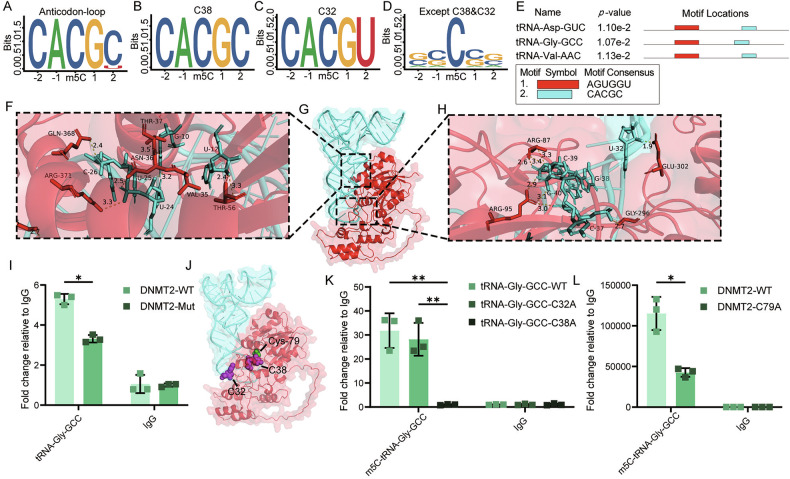
Mechanism of DNMT2 involvement in tRNA m5C methylation modification. **A**–**D** Motif analysis of m5C site upstream and downstream sequences in anticodon-loop (**A**), tRNA C38 (**B**), tRNA C32 (**C**) and other sites except C38 and C32 (**D**). **E** Motif analysis of three tRNA methylated by DNMT2. **F**–**H** Molecular docking of tRNA-Gly-GCC (blue) and DNMT2 (red). **I** RNA immunoprecipitation assay to assess the binding capacity of tRNA-Gly-GCC before and after mutations in predicted binding sites of DNMT2 (*n* = 3, Two-way ANOVA) **J** Representative images from molecular docking of tRNA-Gly-GCC (blue) and DNMT2 (red). The sites where m5C occurs are shown in purple. The sites where DNMT2 activates m5C occurs are shown in green. **K** MeRIP-qPCR determined the methylation level of tRNA-Gly-GCC in tRNA-Gly-GCC-WT cells, tRNA-Gly-GCC-C32A cells and tRNA-Gly-GCC-C38A cells. (*n* = 3, Two-way ANOVA) **L** MeRIP-qPCR determined the methylation level of tRNA-Gly-GCC in DNMT2-WT cells and DNMT2-C79A cells. (*n* = 3, Two-way ANOVA) All the data are shown as the mean ± SD. *P < 0.05; **P < 0.01.

To analyze the reasons why DNMT2 specifically methylates C38 of tRNA-Asp-GUC, tRNA-Gly-GCC and tRNA-Val-AAC, we performed motif analysis on the tRNA sequence of enriched m5C sites. The motif sequences of m5C occurring in the anticodon-loop were mostly CACGC, with only a small portion being CACGU (Fig. 4A). Notably, the motif sequence of all m5C occurring at the C38 site was CACGC (Fig. 4B), while the motif sequence of all m5C occurring at the C32 site was CACGU (Fig. 4C). The motif sequences of m5C occurring at other sites were completely different (Fig. 4D). This suggested that CACGC may serve as a specific recognition sequence required for DNMT2-mediated m5C modification. At the same time, we observed that the motif sequence of the C32 site was only different from that of the C38 site in the last position, which may be the reason why a small amount of m5C modification occurred at the C32 site of tRNA-Gly-GCC. Next, we analyzed the motif sequences of tRNA-Asp-GUC, tRNA-Gly-GCC and tRNA-Val-AAC. In addition to the CACGC sequence, all three tRNAs had another motif sequence AGUGGU (Fig. 4E).

Next, we selected tRNA-Gly-GCC from the three tRNAs for further study. At present, the specific binding sites between DNMT2 and tRNA remain unknown. In order to further explore the mechanism by which DNMT2 activates m5C methylation of tRNA-Gly-GCC, we used SwissModel [16] to perform homology modeling on DNMT2 and AlphaFold3 [17] to predict the structure of tRNA-Gly-GCC. The binding between DNMT2 and tRNA-Gly-GCC was predicted by AlphaFold3 and visualized using PyMOL [18] (Fig. 4F–H). We verified the binding of DNMT2 and tRNA-Gly-GCC using RIP experiments and found that the binding ability of DNMT2 to tRNA-Gly-GCC was significantly reduced when the predicted binding site was mutated (Fig. 4I). We searched the UniProt database (https://www.uniprot.org/uniprotkb/O14717) for the activation site of DNMT2 (Cys79) and visualized the activation site of DNMT2 and the sites where m5C modification occurred in the sequenced tRNA using PyMOL [18] (Fig. 4J). The results showed that compared with C32 site of tRNA-Gly-GCC, Cys79 of DNMT2 could more effectively contact and activate the C38 site. The results of MeRIP-qPCR further supported this observation. After mutating C38 in tRNA-Gly-GCC, the enrichment level of m5C-tRNA-Gly-GCC decreased significantly, whereas mutating C32 caused only a slight decrease that was not significantly different from the wild-type group (Fig. 4K). At the same time, mutating Cys79 markedly reduced the ability of DNMT2 to catalyze m5C modification on tRNA-Gly-GCC (Fig. 4L).

### Downregulation of DNMT2 leads to the ANG-dependent cleavage of tRNA into 5’tiRNA^Gly-GCC^

**Fig. 5:**
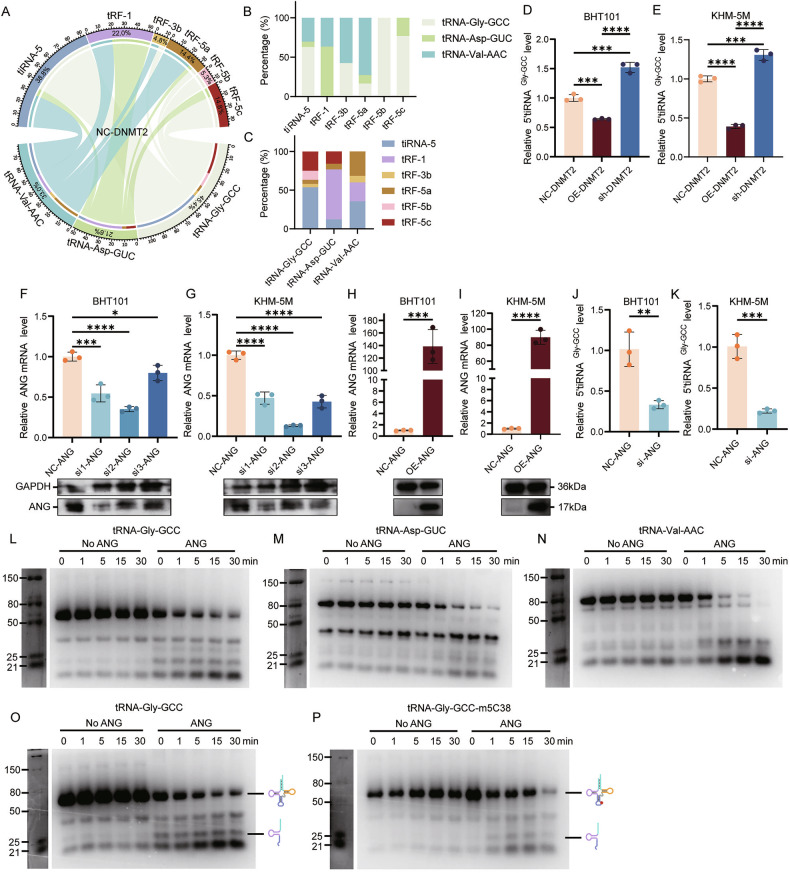
DNMT2 regulates tRFs and tiRNAs generation by affecting m5C levels. **A** Chord diagram presenting the source and proportion of tRFs and tiRNAs in NC-DNMT2 group. **B** Bar graph showing the proportion of three tRNA-derived tRFs and tiRNAs in different tRFs and tiRNAs class. **C** Bar graph showing the proportion of each derived tRFs and tiRNAs in the three tRNA. **D**, **E** RT-qPCR detecting 5’tiRNA^Gly-GCC^ levels of NC-DNMT2, sh-DNMT2 and OE-DNMT2 group in BHT101 cells (**D**) and KHM-5M cells (**E**). (*n* = 3, one-way ANOVA). **F**–**I** RT-qPCR and western blot showing the transfection efficiency of si-ANG (*n* = 3, one-way ANOVA) and oe-ANG (n = 3, Student’s *t*-test) in BHT101 and KHM-5M. **J**, **K** RT-qPCR indicating the change of 5’tiRNA^Gly-GCC^ levels with transfering si-ANG in BHT101 (**J**) and KHM-5M (**K**) cells. (*n* = 3, Student’s *t*-test). **L**–**N** Gel plot of tRNA-Gly-GCC (**L**), tRNA-Asp-GUC (**M**) and tRNA-Val-AAC (**N**) with 2.5 µM ANG and without ANG. (*n* = 3). **O**, **P** Gel plot of tRNA-Gly-GCC (**O**) and tRNA-Gly-GCC-m5C38 (**P**) with 2.5 µM ANG and without ANG. (*n* = 3) All the data are shown as the mean ± SD. *P < 0.05; **P < 0.01; ***P < 0.001; ****P < 0.0001.

The m5C modification helps maintain the stability of tRNA and protects it from being cleaved into tsRNAs by various hydrolases (Supplementary Fig. 3A) [19]. In the previous sections we have shown that DNMT2 is downregulated in ATC cells, resulting in reduced m5C modification levels on tRNA. We therefore speculate that this leads to the cleavage of tRNA into tsRNAs. To prove this hypothesis, we performed tsRNAs sequencing on NC-DNMT2 cells and sh-DNMT2 cells. Since DNMT2 specifically methylates tRNA-Asp-GUC, tRNA-Gly-GCC and tRNA-Val-AAC in ATC cells, we focused our analysis on tsRNAs derived from these three tRNAs. We found that most tsRNAs originated from tRNA-Gly-GCC (45.4% in the NC-DNMT2 group and 44.2% in the sh-DNMT2 group), and tiRNA-5 accounted for the largest proportion (38.8% in the NC-DNMT2 group and 38.3% in the sh-DNMT2 group) (Fig. 5A–C; Supplementary Fig. 3B–D). Notably, tiRNA-5 primarily originates from tRNA-Gly-GCC, followed by tRNA-Val-AAC, and rarely arises from tRNA-Asp-GUC (Fig. 5B, C; Supplementary Fig. 3C, D). Compared with the NC-DNMT2 group, tiRNA-1:31-Gly-GCC (hereafter referred to as 5’tiRNAGly-GCC) was significantly upregulated in the sh-DNMT2 group (Supplementary Fig. 3E). RT-qPCR experiments also showed that knocking down DNMT2 could increase the level of 5’tiRNA^Gly-GCC^, while overexpressing DNMT2 inhibited the generation of 5’tiRNA^Gly-GCC^ in ATC cells (Fig. 5D, E).

Previous studies have found that angiogenin (ANG) can cleave the anticodon-loop of tRNA to generate tiRNA-3 and tiRNA-5 [20]. Since 5’tiRNA^Gly-GCC^ belongs to tiRNA-5, we speculate that it is generated by ANG through cleavage of the anticodon loop of tRNA-Gly-GCC. To test this hypothesis, we knocked down or overexpressed ANG in ATC cells and verified the efficiency (Fig. 5F–I). The level of 5’tiRNA^Gly-GCC^ was significantly decreased after ANG knockdown (Fig. 5J, K). Next, we used in vitro enzyme digestion experiments to detect the cleavage ability of ANG on different tRNAs and to verify the effect of m5C modification on ANG cleavage ability. Our results showed that, in vitro, ANG had a stronger cleavage ability on tRNA-Gly-GCC (Fig. 5L) and tRNA-Val-AAC (Fig. 5N) than tRNA-Asp-AAC (Fig. 5M). This finding is consistent with the aforementioned tsRNA sequencing results, namely that the vast majority of tiRNA-5 generated by ANG cleavage originates from tRNA-Gly-GCC and tRNA-Val-AAC (Fig. 5B; Supplementary Fig. 3C). In addition, m5C38 methylation modification on tRNA can significantly protect tRNA from ANG cleavage (Fig. 5O, P).

**Fig. 6:**
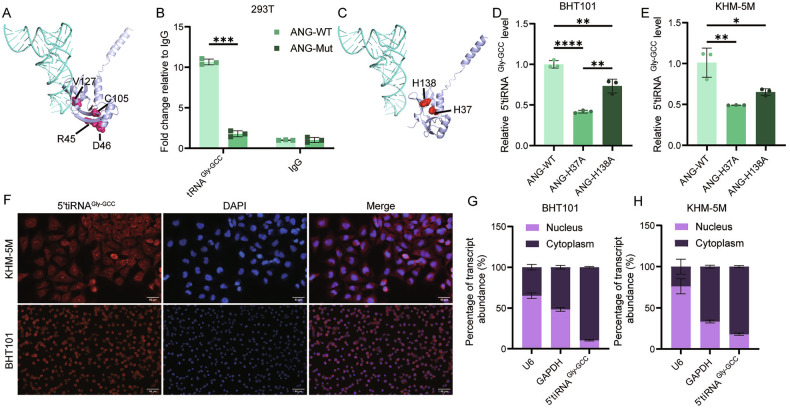
5’tiRNA^Gly-GCC^ is generated by ANG cleavage of tRNA. **A** Representative images from molecular docking of tRNA-Gly-GCC (blue) and ANG (purple). The sites where ANG binds to tRNA are marked in pink. **B** RNA immunoprecipitation assay showing the difference in tRNA-Gly-GCC enrichment levels between ANG-WT group and ANG-mut group in 293T cells. (*n* = 3, Two-way ANOVA). **C** Representative images from molecular docking of tRNA-Gly-GCC (blue) and ANG (purple). **D**, **E** The active sites of ANG are marked in red. RT-qPCR showing 5’tiRNA^Gly-GCC^ levels of BHT101 (**D**) and KHM-5M (**E**) in ANG-WT group, ANG-H37A group and ANG-H138A group. (*n* = 3, One-way ANOVA). **F** Representative images from fluorescence in situ hybridization of 5’tiRNA^Gly-GCC^ in BHT101 cells (down) and KHM-5M cells (up). Red colors indicate 5’tiRNA^Gly-GCC^ and blue colors indicate nucleus. Scale bars, 50 μm. **G**, **H** Nucleocytoplasmic separation and RT-qPCR indicating 5’tiRNA^Gly-GCC^ localization in BHT101 cells (**G**) and KHM-5M cells (**H**). (*n* = 3). All the data are shown as the mean ± SD. *P < 0.05; **P < 0.01; ***P < 0.001; ****P < 0.0001.

Next, we tried to explore the molecular mechanism by which ANG cleaves tRNA-Gly-GCC. We searched for the binding sites of ANG binding to tRNA and the active sites of tRNA hydrolysis in the UniProt database (https://www.uniprot.org/uniprotkb/P03950). SwissModel [16] was used to perform homology modeling of ANG, and the identified sites were visualized on the structural model using PyMOL [18] (Fig. 6A, C). RNA immunoprecipitation (RIP) results showed that mutation of the ANG binding site significantly reduced the enrichment level of tRNA-Gly-GCC (Fig. 6B). For active sites His138 and His37, although mutations in both significantly downregulated the level of 5’tiRNA^Gly-GCC^, the effect of His37 was greater. This suggests that His37 may be the main active site for ANG to cleave tRNA-Gly-GCC to generate 5’tiRNA^Gly-GCC^ in ATC cells (Fig. 6D, E). Meanwhile, 5’tiRNA^Gly-GCC^ was present in both the cytoplasm and nucleus in ATC cells, but was mainly localized in the cytoplasm, as demonstrated by immunofluorescence staining (Fig. 6F) and nucleocytoplasmic separation (Fig. 6G, H).

### 5’tiRNA^Gly-GCC^ is highly expressed in ATC cells and promotes ATC progression

**Fig. 7:**
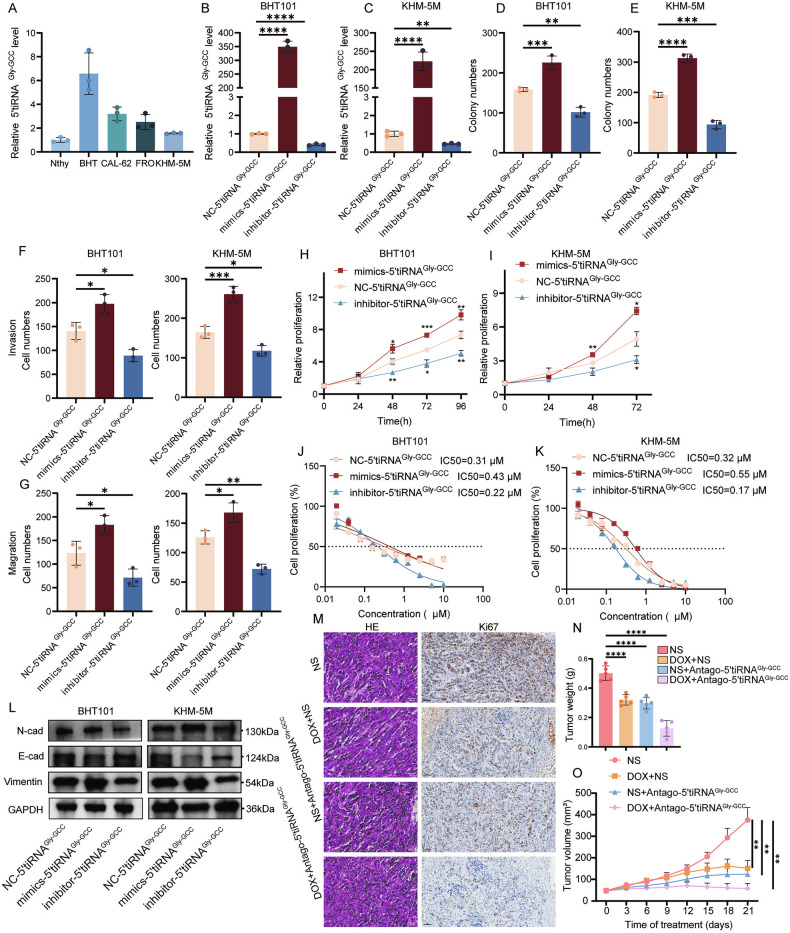
High levels of 5’tiRNA^Gly-GCC^ promote the progression of anaplastic thyroid cancer. **A** RT-qPCR showing 5’tiRNA^Gly-GCC^ levels in normal thyroid cell line and ATC cell lines. (*n* = 3). **B**, **C** RT-qPCR analysis the transfection efficiency of mimics-5’tiRNA^Gly-GCC^ and inhibitor-5’tiRNA^Gly-GCC^ in BHT101 (**B**) and KHM-5M (**C**). (*n* = 3, one-way ANOVA). **D**, **E** Colony-forming assays to evaluate the impacts of mimics-5’tiRNA^Gly-GCC^ or inhibitor-5’tiRNA^Gly-GCC^ in BHT101 (**D**) and KHM-5M cells (**E**). (*n* = 3, one-way ANOVA). **F**, **G** Statistical analysis of transwell assays to show the invasion (**F**) and migration (**G**) abilities after transferring mimics-5’tiRNA^Gly-GCC^ or inhibitor-5’tiRNA^Gly-GCC^ in BHT101 and KHM-5M cells. (*n* = 3, one-way ANOVA). **H**, **I** Cell viability measured by CCK8 assay after transferring mimics-5’tiRNA^Gly-GCC^ or inhibitor-5’tiRNA^Gly-GCC^ in BHT101 (**H**) and KHM-5M (**I**) cells with different times (0, 24, 48, 72, 96 h). (*n* = 3, two-way ANOVA). **J**, **K** IC50 values of doxorubicin HCl in BHT101 (**J**) and KHM-5M (**K**) cells in NC-5’tiRNA^Gly-GCC^, mimics-5’tiRNA^Gly-GCC^ and inhibitor-5’tiRNA^Gly-GCC^. (*n* = 3). **L** Western blot to analyze the EMT pathway related proteins expression levels in BHT101 (right) and KHM-5M (left) cells. (*n* = 3). **M** Representative images from H&E staining (Scale bars, 100 μm) and Ki-67 IHC staining (Scale bars, 50 μm) for tumor tissue in each group. **N** Tumor weights comparison in each group. (*n* = 5, one-way ANOVA). **O** Tumor growth curves of each group. (*n* = 5, two-way ANOVA). All the data are shown as the mean ± SD. *P < 0.05; **P < 0.01; ***P < 0.001; ****P < 0.0001.

To further explore the role of 5’tiRNA^Gly-GCC^ in ATC cells, we investigated how 5’tiRNA^Gly-GCC^ influences the ATC cellular phenotype. In both normal thyroid cell lines and ATC cell lines, the results showed that 5’tiRNA^Gly-GCC^ was highly expressed in ATC cells (Fig. 7A). Next, we selected BHT101 and KHM-5M cell lines to further overexpress and inhibit 5’tiRNA^Gly-GCC^ (Fig. 7B, C). Cloning experiments (Fig. 7D, E; Supplementary Fig. 4A) and CCK8 assays (Fig. 7H, I) indicated that mimics-5’tiRNA^Gly-GCC^ facilitated the proliferation of ATC cells, whereas inhibitor-5’tiRNA^Gly-GCC^ suppressed it. Transwell experiments further demonstrated that increased 5’tiRNA^Gly-GCC^ levels can enhance the invasion and metastasis potential of ATC cells (Fig. 7F, G; Supplementary Fig. 4B). At the same time, we found that 5’tiRNA^Gly-GCC^ levels can regulate the sensitivity of ATC cells to doxorubicin hydrochloride. Specifically, mimics-5’tiRNA^Gly-GCC^ increased the IC50 of doxorubicin hydrochloride, while inhibitor-5’tiRNA^Gly-GCC^ produced the opposite result (Fig. 7J, K). Western blot analysis of EMT pathway proteins showed that mimics-5’tiRNA^Gly-GCC^ activated the EMT pathway, while inhibitor-5’tiRNA^Gly-GCC^ suppressed EMT activation (Fig. 7L). Meanwhile, 5’tiRNA^Gly-GCC^ partially rescued the effects of DNMT2 on ATC proliferation (Supplementary Fig. 5B, C), invasion and migration (Supplementary Fig. 4F, G; Supplementary Fig. 5A), as well as the EMT pathway (Supplementary Fig. 5D).

Since high expression of 5’tiRNA^Gly-GCC^ can effectively promote ATC cell progression and drug resistance in vitro, we further explored the possibility of targeting 5’tiRNA^Gly-GCC^ treatment of ATC in vivo (Supplementary Fig. 4C, D). The results showed that Antago-5’tiRNA^Gly-GCC^ had a similar effect to doxorubicin hydrochloride in inhibiting the growth of tumors subcutaneously loaded ATC cells in nude mice. It is worth noting that the combination of Antago-5’tiRNA^Gly-GCC^ and doxorubicin hydrochloride performed significantly better than either alone, as reflected in tumor volume (Fig. 7O), tumor weight (Fig. 7N), and Ki67 levels (Fig. 7M). At the same time, no obvious drug toxicity was observed in nude mice (Supplementary Fig. 4E).

### 5’tiRNA^Gly-GCC^ binds to hnRNPH1 to exert oncogenic effects

**Fig. 8:**
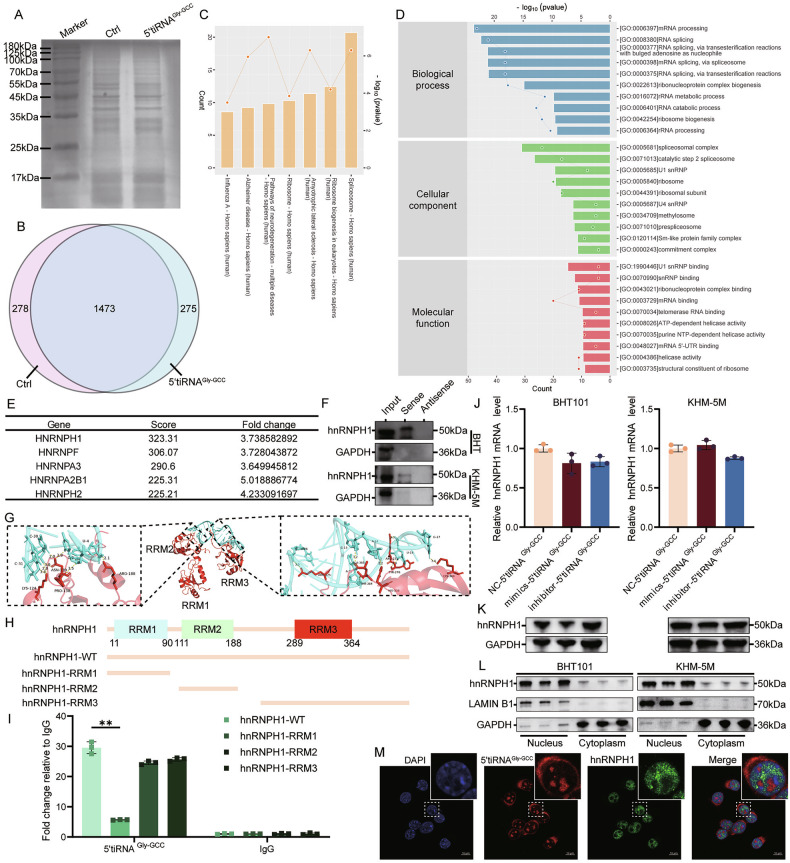
5’tiRNAGly-GCC binds to hnRNPH1 to exert oncogenic effects. **A**–**D** RNA pulldown assay followed by silver staining (**A**) and CHIRP-MS showing protein enrichment summary of 5’tiRNA^Gly-GCC^ with venn diagram (**B**), KEGG enrichment analysis (**C**) and GO enrichment analysis (**D**). **E** The table shows the top five 5’tiRNA^Gly-GCC^ -specific binding proteins with the highest scores. **F** RNA pulldown assay and western blot proving the binding of 5’tiRNA^Gly-GCC^ and hnRNPH1 in BHT101 cells (up) and KHM-5M cells (down). (*n* = 3) **G** Representative images from molecular docking of 5’tiRNAGly-GCC (blue) and hnRNPH1 (red). **H** Schematic diagram of hnRNPH1 full-length and domain-specific truncated mutations. **I** RNA immunoprecipitation assay detecting the binding ability of 5’tiRNAGly-GCC and hnRNPH1-WT and each domain-specific truncated mutations. (*n* = 3, two-way ANOVA). **J**, **K** RT-qPCR (**J**) and western blot (**K**) showing hnRNPH1 levels of NC-5’tiRNAGly-GCC, mimics-5’tiRNAGly-GCC and inhibitor-5’tiRNAGly-GCC group in BHT101 cells (left) and KHM-5M cells (right). (*n* = 3, one-way ANOVA). **L** Nucleocytoplasmic separation and western blot indicating the effect of 5’tiRNAGly-GCC binding on the cellular localization and expression level of hnRNPH1 in BHT101 cells (left) and KHM-5M cells(right). (*n* = 3). **M** Immunofluorescence analysis and representative confocal microscopy for 5’tiRNAGly-GCC (red) and hnRNPH1 (green). Blue colors indicate nucleus. Scale bars, 10 μm. All the data are shown as the mean ± SD. **P < 0.01.

Numerous studies have shown that tsRNAs exert their effects by binding to RNA-binding protein (RBP) [21, 22]. To investigate this, we performed silver staining after RNA pulldown experiment (Fig. 8A) and CHIRP-MS in KHM-5M cell line. The results showed that 5’tiRNA^Gly-GCC^ specifically bound to 275 RBPs (Fig. 8B). KEGG (Fig. 8C) and GO enrichment analysis (Fig. 8D) of these 275 proteins revealed that 5’tiRNA^Gly-GCC^ mainly bound to proteins related to RNA alternative splicing. These 275 RBPs were sorted by score, and hnRNPH1 with the highest score was selected for further study (Fig. 8E). We then performed RNA-pulldown experiments again in BHT101 cell lines to confirm the interaction between 5’tiRNA^Gly-GCC^ and hnRNPH1 (Fig. 8F, M). At the same time, we found that overexpression or inhibition of 5’tiRNA^Gly-GCC^ levels did not change the mRNA level of hnRNPH1 (Fig. 8J), but markedly affected the protein level. Specifically, mimics-5’tiRNA^Gly-GCC^ inhibited the protein level of hnRNPH1 while inhibitor-5’tiRNA^Gly-GCC^ promoted it (Fig. 8K). Correlation analysis of ATC data from GSE65144 and GSE33630, as well as TC data from TCGA, demonstrated a significant positive correlation between DNMT2 and hnRNPH1 (Supplementary Fig. 6A). In addition, CIBERSORT analysis of these data revealed that hnRNPH1 was negatively correlated with immune-infiltrating cells, especially Tregs cells (Supplementary Fig. 6B, C), consistent with the CIBERSORT results of DNMT2. Nucleocytoplasmic separation and western blot showed that the binding of 5’tiRNA^Gly-GCC^ and hnRNPH1 only altered the protein level of hnRNPH1 but did not affect its subcellular localization (Fig. 8L). We further used SwissModel [16] to perform homology modeling on hnRNPH1 and used AlphaFold3 [17] to predict its binding with 5’tiRNA^Gly-GCC^ which was visualized by PyMOL [18]. The results showed that 5’tiRNA^Gly-GCC^ mainly bound to RRM2 and RRM3 domains of hnRNPH1(Fig. 8G). To prove this, we constructed truncating mutations of RRM1, RRM2 and RRM3 of hnRNPH1 and performed RIP (Fig. 8H). The RIP results showed that compared with the hnRNPH1-WT group, the 5’tiRNA^Gly-GCC^ enriched in the RRM2 or RRM3 domain decreased slightly but no significant difference, only the 5’tiRNA^Gly-GCC^ enriched in RRM1 decreased significantly (Fig. 8I).

Study has found that hnRNPH1 can bind to and negatively regulate PD-L1 transcripts in glioblastoma [23]. Considering that PD-L1 is highly expressed in most ATC patients [24, 25], we speculate that 5’tiRNA^Gly-GCC^ upregulates PD-L1 by inhibiting hnRNPH1. We analyzed the ATC cohort from the GEO database and found a negative correlation between DNMT2 and PD-L1 expression (Supplementary Fig. 7A). Next, we knocked down and overexpressed hnRNPH1 in BHT101 and KHM-5M cell lines and verified the efficiency (Supplementary Fig. 7C-F). Western blotting results showed that PD-L1 protein levels significantly increased after hnRNPH1 knockdown, while overexpression of hnRNPH1 showed the opposite result (Supplementary Fig. 7G). The same results were observed after DNMT2 knockdown and overexpression (Supplementary Fig. 7H). Furthermore, the changes in PD-L1 protein levels after 5’tiRNA^Gly-GCC^ inhibition or overexpression were the opposite of those after hnRNPH1 and DNMT2 (Supplementary Fig. 7I). Simultaneously, the PD-L1 level changes caused by DNMT2 could be rescued by 5’tiRNA^Gly-GCC^ (Supplementary Fig. 7J). Many studies have shown that PD-L1 can promote Tregs infiltration and enhance the immunosuppressive function of Tregs [26, 27]. This is consistent with our CIBERSORT results, which show that in TC, both DNMT2 and hnRNPH1 are negatively correlated with Treg cells (Supplementary Fig. 1B, Supplementary Fig. 6B), while PD-L1 is positively correlated with Tregs (Supplementary Fig. 7B). Furthermore, Tregs have higher CIBERSORT scores in TC (Supplementary Fig. 1D).

## Discussion

Many studies have reported aberrant m5C modification during the initiation and progression of multiple tumors [9, 28, 29]. As a confirmed m5C methyltransferase, DNMT2 has been observed to be highly expressed in various tumors, including hepatocellular carcinoma, osteosarcoma, glioblastoma, and so on [30–32]. In addition, DNMT2 is regarded as a promising therapeutic target in a variety of tumors, as its knockdown can promote the clearance of cancer cells during chemotherapy [11]. However, our previous work demonstrated that DNMT2 is downregulated in ATC [12]. The results of this paper also showed that low levels of DNMT2 contribute to the occurrence, development, and drug resistance of ATC cells. Moreover, DNMT2 appears to act as a protective factor for ATC and is not conducive to the infiltration of immunosuppressive cells. This suggests that DNMT2 has an unknown mechanism for regulating tumor progression in ATC.

This study showed that in ATC, DNMT2 specifically methylates the m5C site at C38 of tRNA-Asp-GUC, tRNA-Gly-GCC, and tRNA-Val-AAC. This is consistent with the DNMT2 substrates that have been demonstrated so far. Nucleophilic attack residue C79 of DNMT2 is strictly conserved, which determines the consistent catalytic mechanism of DNMT2 [33]. This is also observed by MeRIP-qPCR in this paper. The C32U33(G/I)34N35 (C/U)36A37C38 motif in the anticodon loop is considered to be necessary for DNMT2 to recognize and methylate tRNA [34]. Through MeRIP-seq, we found that all DNMT2-specifically methylated tRNAs in ATC cells have the CACGC motif, which also reflects the conserved recognition and activation mechanism of DNMT2 and tRNA in mammals.

m5C methylation modification plays a critical role in preserving tRNA integrity by preventing its fragmentation into tsRNAs [35]. According to the different cleavage sites, tsRNAs are generally classified into two major types: tiRNAs and tRFs, and their generation is not random but tightly regulated by specific nucleases. In ATC cells, we observed that the predominant tsRNA species were tiRNA-5, whose cleavage occurs within the anticodon loop. We speculate that this is because the m5C modification of tRNA mediated by DNMT2 occurs at C38 of the anticodon-loop, whose downregulation leads to decreased stability of the anticodon-loop and makes it more susceptible to fragmentation. The generation of tiRNAs is mostly activated by ANG, which cleaves the anticodon loop of tRNA [36]. Current studies have shown that ANG binds to tRNA through R45, D46, V127, and C105 and activates the cleavage reaction through H138 and H37. In this study, we also verified these sites by RIP and RT-qPCR. Interestingly, our results showed that most tiRNA-5s were derived from tRNA-Gly-GCC, and only a small part of tiRNAs were derived from tRNA-Asp-GUC. We speculated that this difference might be a combination of the following factors: (a) the different base sequences of the three tRNA-Asp-GUC, tRNA-Gly-GCC, and tRNA-Val-AAC lead to different affinities of ANG for these three tRNAs, and (b) the stability of tiRNA-5 from different tRNAs is different. Studies have found that overexpression of ANG can lead to the cleavage of specific tRNAs, including tRNA-Asp-GUC, tRNA-Gly-GCC, and tRNA-Val-AAC [37]. At the same time, tiRNA-5 derived from tRNA-Gly-GCC has better stability. These findings also confirm our speculation [38].

There is an active crosstalk between dysregulation of tsRNAs and oncogenes, and it is considered a useful marker for cancer diagnosis or a potential target for treatment [39–41]. In this article, we also observed that downregulation of DNMT2 led to high expression of 5’tiRNA^Gly-GCC^, which effectively promoted the proliferation, invasion, and metastasis of ATC. It also promoted the epithelial-mesenchymal transition (EMT) of ATC. This is consistent with the characteristics of ATC reported so far [42]. The regulatory pathway of EMT is a complex network [43], and the pathway of 5’tiRNA^Gly-GCC^ regulating EMT still needs further exploration. Chemotherapy is one of the key treatments for ATC, but due to its low response rate and high drug resistance in ATC patients, it is currently primarily used for patients without targeted or immunotherapy options, or in combination with other therapies [5, 6]. As the FDA-approved first-line chemotherapy agent for ATC patients, the molecular mechanisms underlying doxorubicin resistance in ATC and strategies for overcoming this resistance represent a major research focus [44]. Previous studies have demonstrated that cancer cells regulate their sensitivity to doxorubicin through various methylation modifications [45–47]. Our prior research also indicates that NSUN2-mediated m5C modification can modulate doxorubicin resistance in ATC [12]. Therefore, we hypothesize that DNMT2-mediated tRNA m5C38 modification is related to doxorubicin resistance. Our results show that low levels of DNMT2 helped ATC cells resist doxorubicin hydrochloride, while inhibiting the level of 5’tiRNA^Gly-GCC^ relieved it. Since DNMT2 is highly expressed in most tumors, the use of DNMT2 activators in ATC patients may lead to the occurrence of other diseases. Therefore, we focus on inhibiting 5’tiRNA^Gly-GCC^ in the direction of finding new targets for ATC. Our results also showed that 5’tiRNA^Gly-GCC^ inhibitor alone or in combination with doxorubicin hydrochloride produced a good inhibitory effect on ATC progression in vivo, and no obvious cytotoxicity was observed.

More and more studies have shown that tsRNA exerts its biological functions through a variety of regulatory mechanisms, including epigenetic regulation, transcriptional or post-transcriptional regulation, and translational regulation [48]. To explore the regulatory mechanism of 5’tiRNA^Gly-GCC^, we performed CHIRP-MS to search for the RBP. The results showed that 5’tiRNA^Gly-GCC^ mainly binds to RRM2 and RRM3 of the alternative splicing protein hnRNPH1, downregulating its protein level without affecting its subcellular localization. Previous studies have demonstrated that hnRNPH1 can downregulate PD-L1 expression [23]. Given that PD-L1 is positively expressed in many ATC patients [25], we hypothesize that the DNMT2-5’tiRNA^Gly-GCC^-hnRNPH1 axis influences PD-L1 protein levels in ATC, thereby regulating disease progression. Our findings above corroborate this hypothesis. It is widely accepted that high PD-L1 expression on tumor cells promotes Treg infiltration and immune tolerance. Immunological analysis of TC cohorts in the TCGA and GEO databases further confirmed the association between the DNMT2-5’tiRNA^Gly-GCC^ -hnRNPH1 axis and Tregs. However, due to the rarity of ATC and the lack of mouse-derived cell lines, we were unable to conduct experiments in clinical samples or animal models to validate the link between the DNMT2 axis and Tregs, or its impact on immune tolerance in ATC. This represents a key direction for our future work.

## Conclusion

In summary, this study revealed that DNMT2 plays an important role in promoting ATC proliferation, invasion, migration and drug resistance by specifically remodeling tRNA m5C methylation. tiRNA generated by ANG cleaving tRNA with low m5C levels exerts oncogenic effects by binding to alternative splicing proteins. These findings provide new directions for revealing the occurrence and development mechanism of ATC and exploring new therapeutic targets.

## Supplementary information

Supplementary material-Supplemental figures

Supplementary Table 1. shRNA and siRNA targeting sequences

Supplemental Table 2. Primer sequences

Supplementary material-Original Blots

## Data Availability

The raw data that support the findings of this study are available from the corresponding author upon reasonable request.

## Code availability

All statistical analysis was performed using GraphPad Prism (v 10.3.1) and R (v 4.3.1). The code used in this study may be obtained from the corresponding author upon reasonable request. We downloaded thyroid cancer tissues and normal tissues data from the TCGA database (https://portal.gdc.cancer.gov). GSE33630 and GSE65144 data were obtained from the GEO database through the GEO query package. Use the limma package to analyze the difference between two groups. The ggplot2 package was used for data visualization.
