# Supplementary material for: DNMT2 inhibits anaplastic thyroid cancer progression by downregulating 5’tiRNAGly-GCC production
Source: Cell Death Dis. 2026 Feb 21;17(1):240. doi: 10.1038/s41419-026-08488-5 (PMC12949022; doi:10.1038/s41419-026-08488-5)
Supplement: Supplementary file 1 — Supplementary material-Supplemental figures [file 41419_2026_8488_MOESM1_ESM.docx]

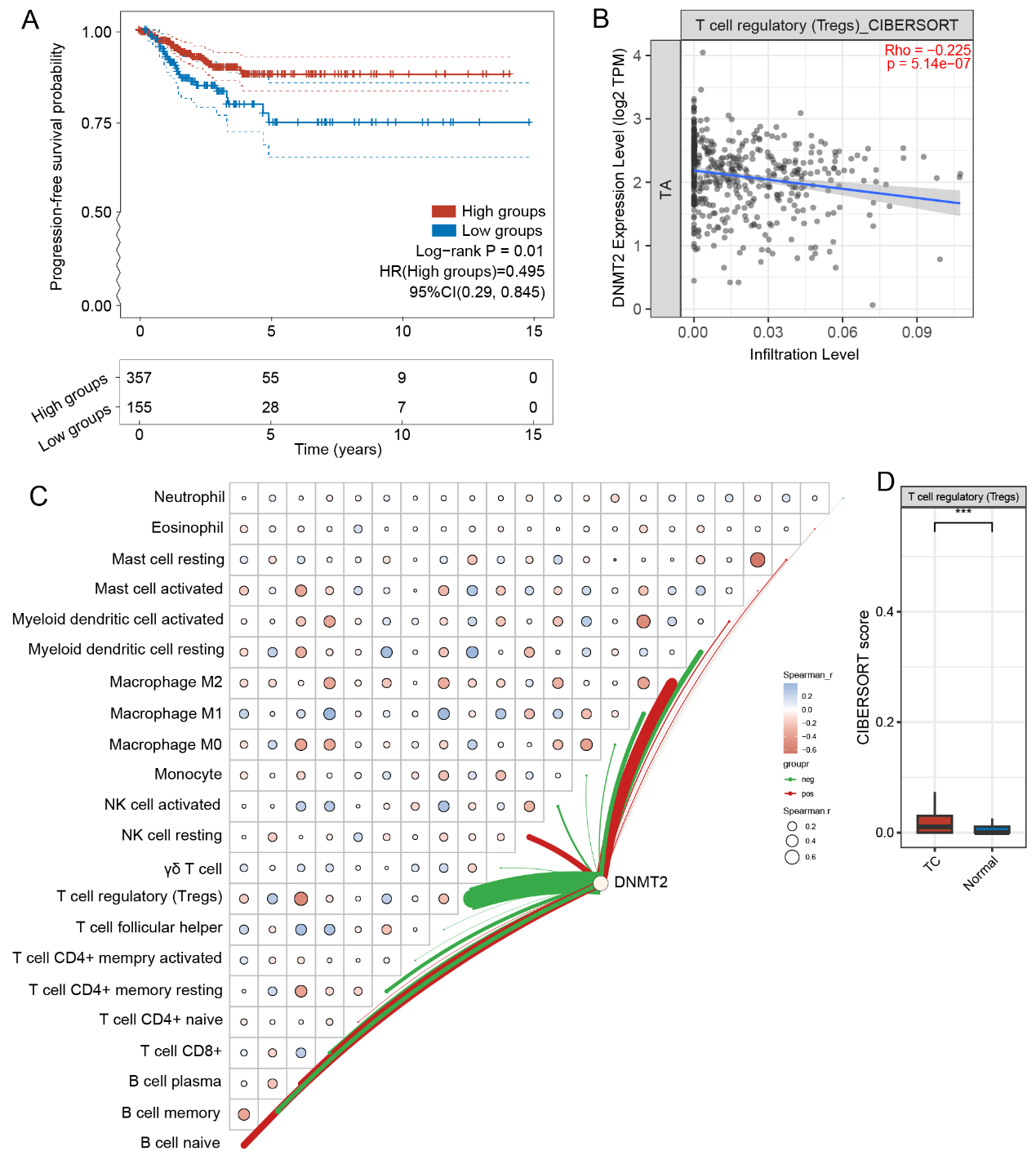


**Supplemental figure1. Effects of DNMT2 on prognosis and immunity of thyroid cancer**

(A) The KM survival curve of PFS of the DNMT2 in thyroid cancer from TCGA data where different groups are tested using the log-rank test. (B) The Spearman correlation analysis plot showing the correlation between DNMT2 and the CIBERSORT immune score of Tregs. (C) The heatmap represents the correlation analysis among the immune scores themselves, where red represents positive correlation and green represents negative correlation. The lines represent the correlation between DNMT2 expression and the CIBERSORT immune scores of different immune cells, where red line indicates a positive correlation and green line indicates a negative correlation. (D) CIBERSORT immune scores of Tregs in thyroid cancer and normal tissues from TCGA data and GEO data. ***P<0.001.


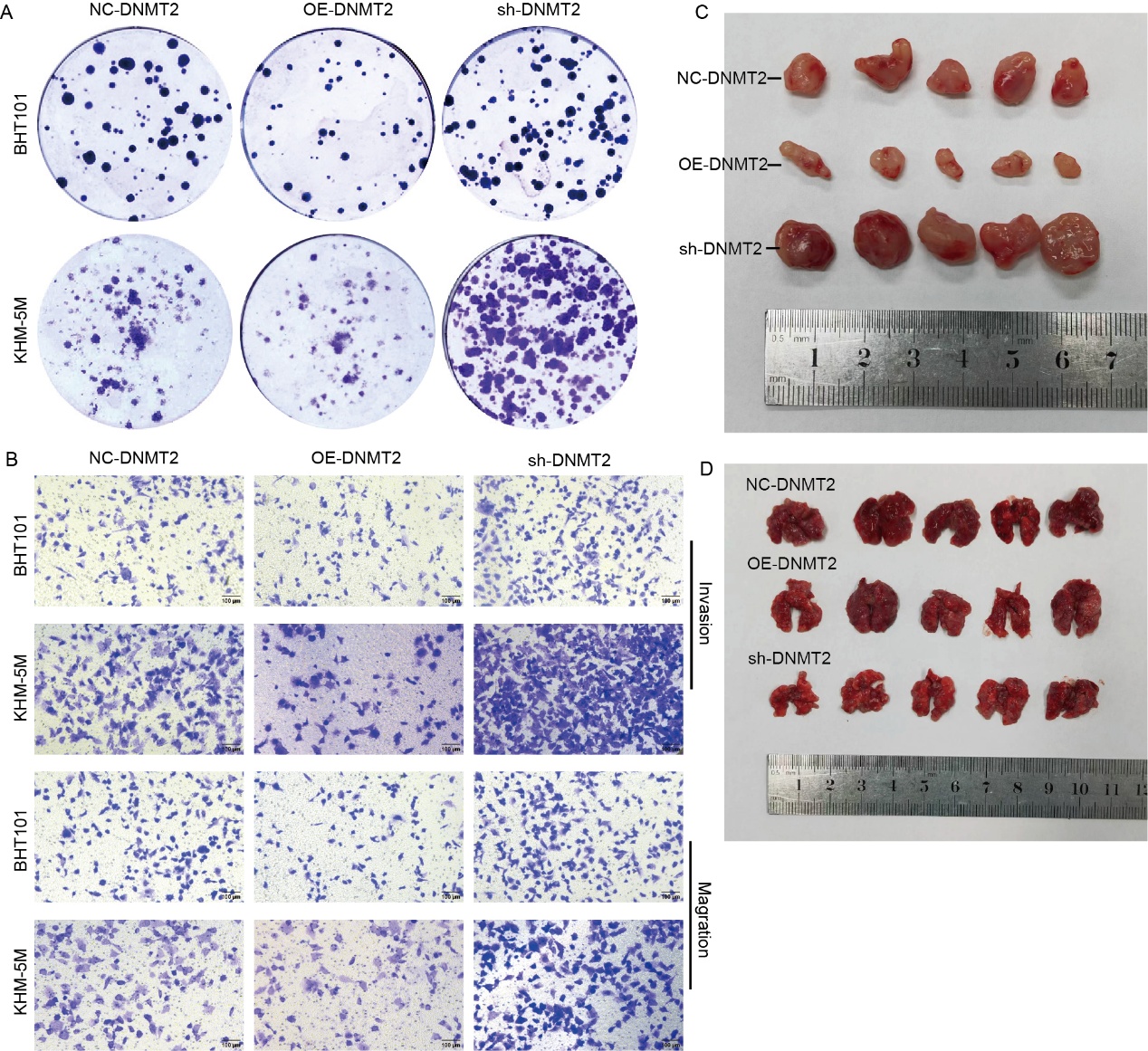


**Supplemental figure2. Representative images of DNMT2 phenotypic analysis.**

(A) Representative images from clone formation ability of BHT101 cells and KHM-5M cells in vitro affected by DNMT2. (B) Representative images from transwell invasion and migration of three groups in BHT101 cells and KHM-5M cells. Scare bars, 100μm. (C) Tumor images on nude mice bearing subcutaneous BHT101 tumor mode. (D) Lung tissues images of metastatic foci in lungs from each group.


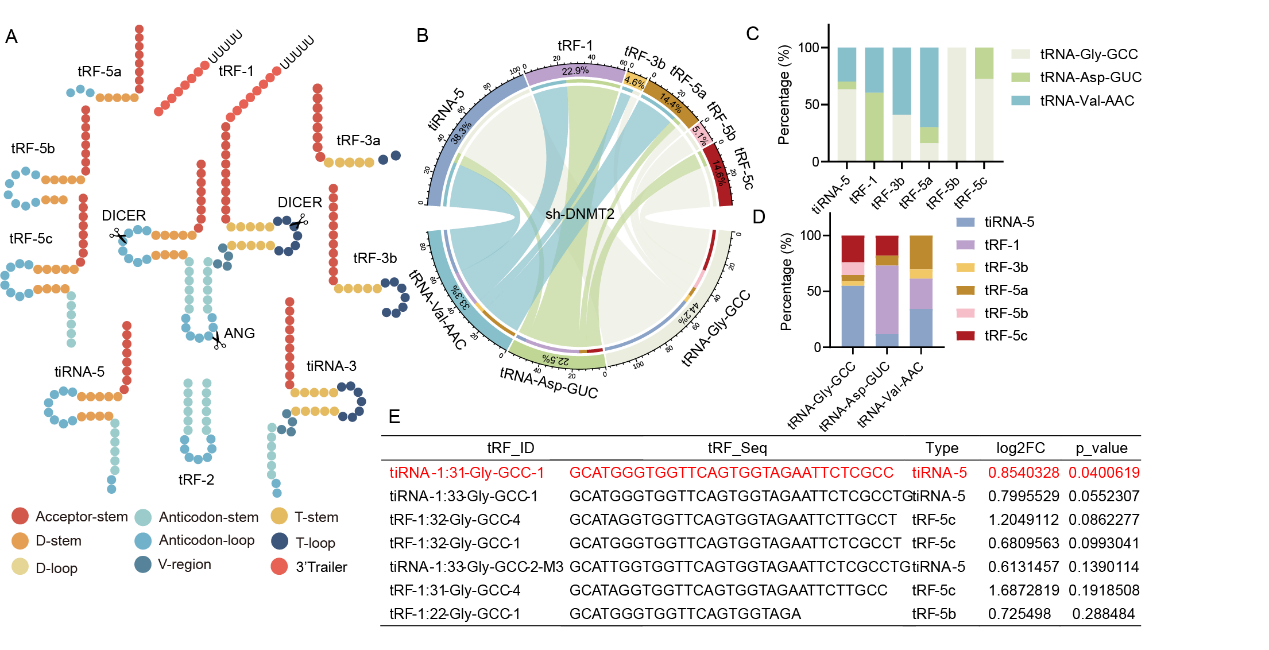


**Supplemental figure 3. tRFs and tiRNAs sequencing results.**

(A) Types of tRFs and tiRNAs. (B) Chord diagram presenting the source and proportion of tRFs and tiRNAs in sh-DNMT2 group. (C) Bar graph showing the proportion of three tRNA-derived tRFs and tiRNAs in different tRFs and tiRNAs class in sh-DNMT2 group. (D) Bar graph showing the proportion of each derived tRFs and tiRNAs in the three tRNA in sh-DNMT2 group. (E) Table showing tRFs and tiRNAs derived from tRNA-Gly-GCC.


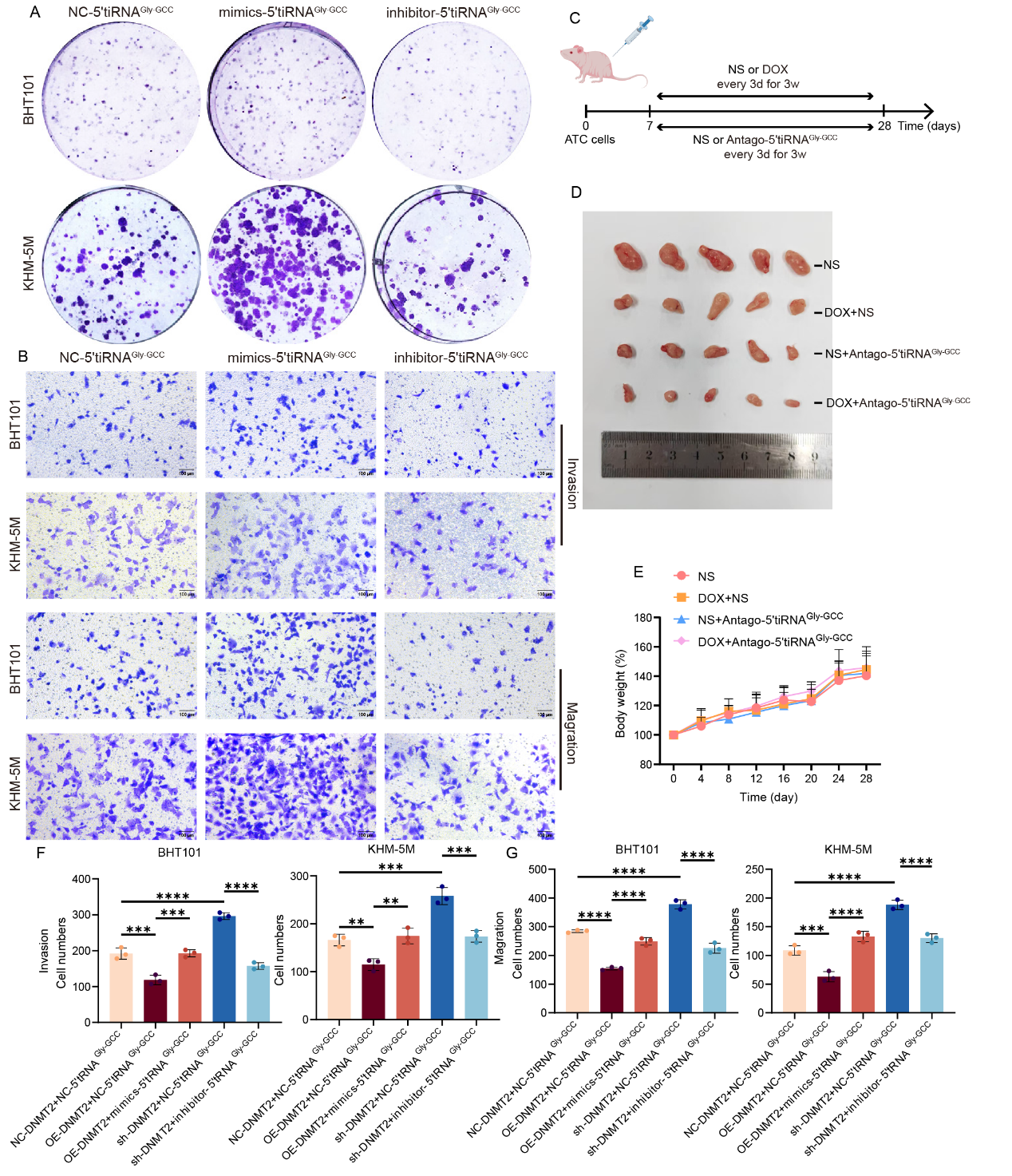


**Supplemental figure 4. 5’tiRNAGly-GCC phenotypic analysis and partial rescue experiment.**

(A) Representative images from clone formation ability of BHT101 cells and KHM-5M cells in vitro affected by 5’tiRNAGly-GCC. (B) Representative images from transwell invasion and migration of three groups in BHT101 cells and KHM-5M cells. Scare bars, 100μm. (C) Drug treatment experimental method in nude mice. (D) Tumor images on nude mice bearing subcutaneous BHT101 tumor mode treated with different drug. (E) Body weight change curve of nude mice after drug treatment. (F-G) Statistical analysis of transwell assays to show the invasion (F) and migration (G) abilities of every groups in BHT101 and KHM-5M cells. (n=3, one-way ANOVA). All the data are shown as the mean ± SD. ***P<0.001; ****P<0.0001.


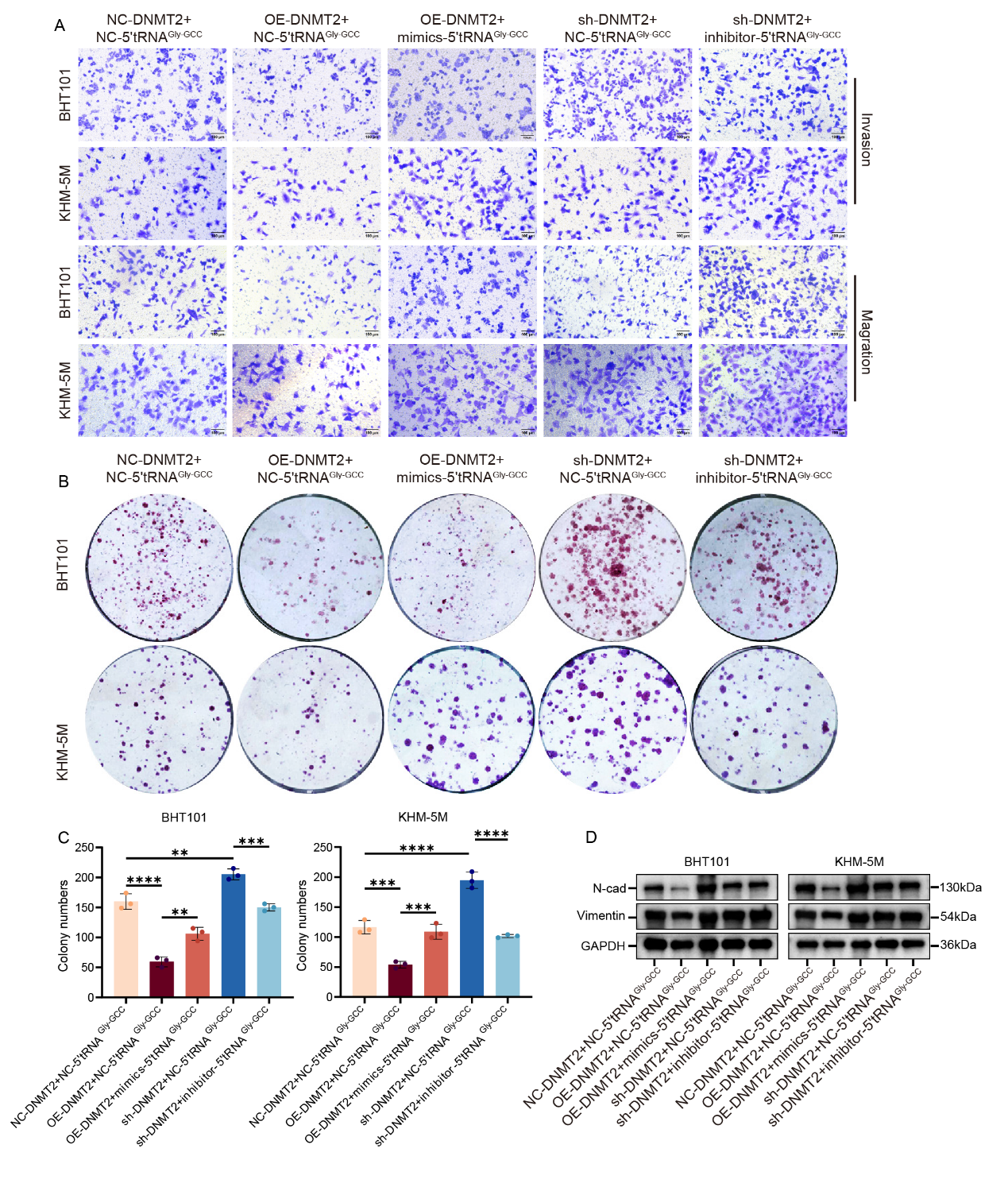


**Supplemental figure5. 5’tiRNAGly-GCC partially rescue the effects of DNMT2.**

(A) Representative images from transwell invasion and migration of five groups in BHT101 cells and KHM-5M cells. Scare bars, 100μm. (B) Representative images from clone formation ability of BHT101 cells and KHM-5M cells in vitro of five groups. (C) Colony-forming assays to evaluate the rescue effect of 5'tiRNAGly-GCC on DNMT2 in BHT101 (left) and KHM-5M (right) cells. (n=3, one-way ANOVA). (D) Western blot evaluating the rescue effect of 5'tiRNAGly-GCC on DNMT2 to EMT pathway. All the data are shown as the mean ± SD. **P<0.01; ***P<0.001; ****P<0.0001.


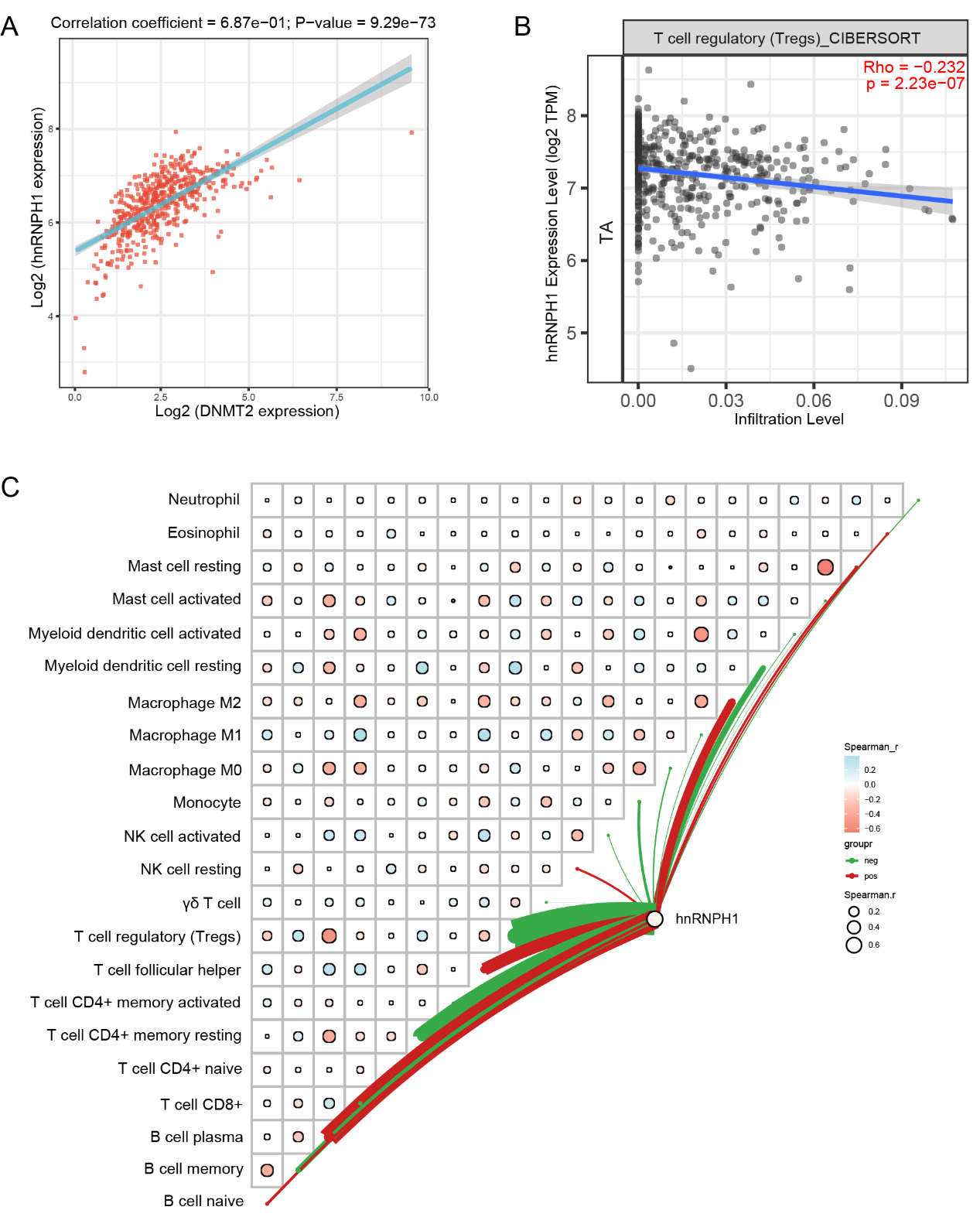


**Supplemental figure6. Correlation analysis between hnRNPH1 and DNMT2 and immune analysis of hnRNPH1.**

(A) Spearman correlation analysis between DNMT2 and hnRNPH1 expression in thyroid cancer from TCGA data and GEO data. (B) The Spearman correlation analysis plot showing the correlation between hnRNPH1 and the CIBERSORT immune score of Tregs. (C) The heatmap represents the correlation analysis among the immune scores themselves, where red represents positive correlation and green represents negative correlation. The lines represent the correlation between hnRNPH1 expression and the CIBERSORT immune scores of different immune cells, where red line indicates a positive correlation and green line indicates a negative correlation.


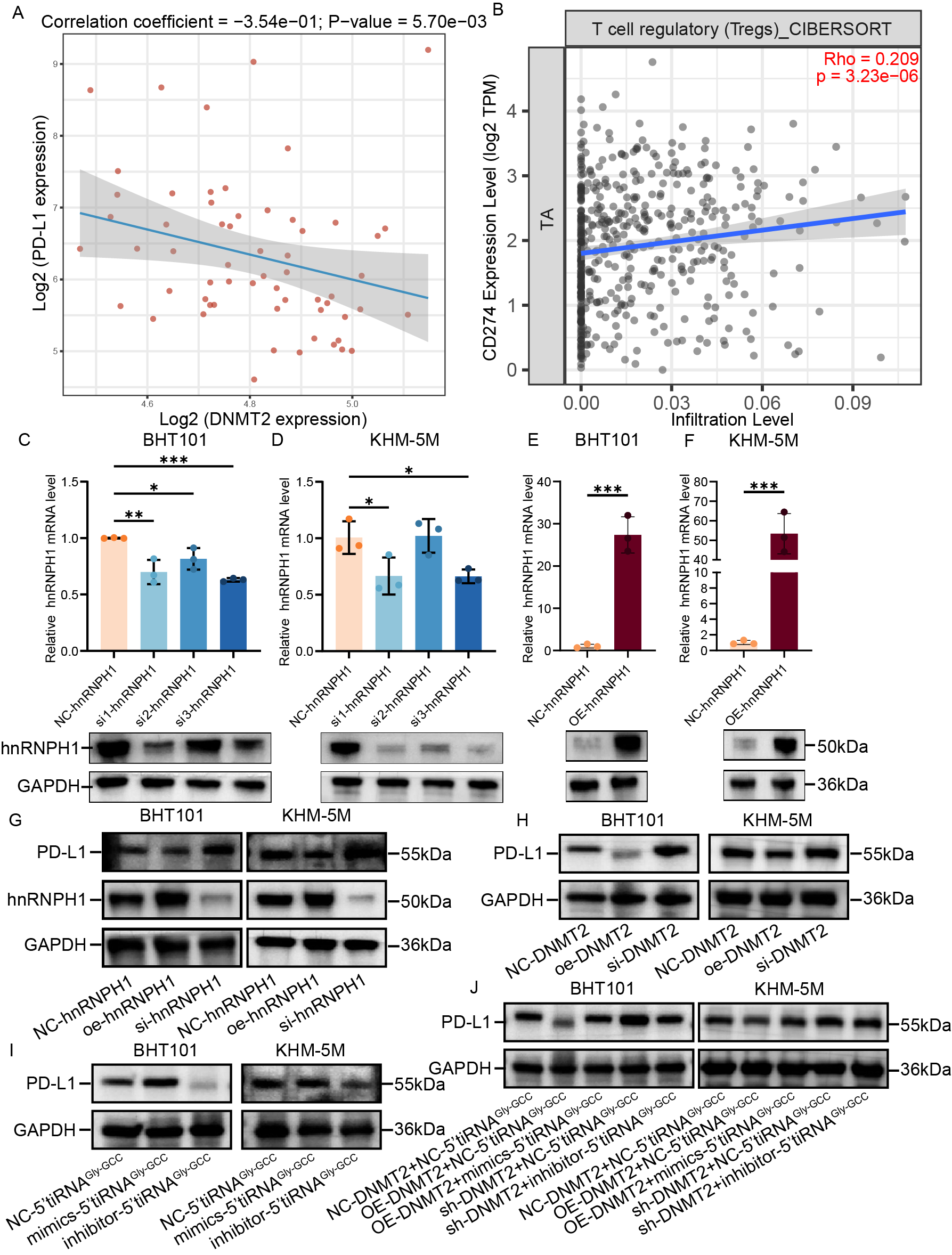


**Supplemental figure7. The DNMT2-5’tiRNA^Gly-GCC^-hnRNPH1 axis regulates the infiltration of Tregs in ATC by modulating PD-L1.**

(A) In ATC data derived from the GEO database, DNMT2 and PD-L1 are negatively correlated. (B) The Spearman correlation analysis plot showing the correlation between PD-L1 and the CIBERSORT immune score of Tregs. (C-D) Verification of hnRNPH1 RNA and protein levels knocked down in BHT101 (C) and KHM-5M (D) cell lines. (n=3, one-way ANOVA) (E-F) Validation of hnRNPH1 overexpression at both the RNA and protein levels in BHT101 (E) and KHM-5M (F) cell lines. (n=3, Student’s t-test) (G) Changes in PD-L1 protein levels after knockdown and overexpression of hnRNPH1 in BHT101 (left) and KHM-5M(right) cell lines. (n=3) (H) Changes in PD-L1 protein levels after knockdown and overexpression of DNMT2 in BHT101 (left) and KHM-5M(right) cell lines. (n=3) (I) Changes in PD-L1 protein levels after knockdown and overexpression of 5’tiRNA^Gly-GCC^ in BHT101 (left) and KHM-5M(right) cell lines. (n=3) (J) Western blot evaluating the rescue effect of 5'tiRNA^Gly-GCC^ on DNMT2 to PD-L1. (n=3) All the data are shown as the mean ± SD. **P<0.01; ***P<0.001.
