# Supplementary material for: DNMT2 inhibits anaplastic thyroid cancer progression by downregulating 5’tiRNAGly-GCC production
Source: Cell Death Dis. 2026 Feb 21;17(1):240. doi: 10.1038/s41419-026-08488-5 (PMC12949022; doi:10.1038/s41419-026-08488-5)
Supplement: Supplementary file 2 — Supplementary Table 1. shRNA and siRNA targeting sequences [file 41419_2026_8488_MOESM2_ESM.docx]

Supplemental Table 1. shRNA and siRNA targeting sequences

| **Gene Name** | **Targeting sequences** |
| --- | --- |
| siANG-1 | GGTCACCACTTGCAAGCTA |
| siANG-2 | GCATCAAGGCCATCTGTGA |
| siANG-3 | GGTTCAGAAACGTTGTTGT |
| sihnRNPH1-1 | CAAAUAUGCAACACAGAUATT |
| sihnRNPH1-2 | GAGAGUACACAUUGAAAUUTT |
| sihnRNPH1-3 | GGAUUACCUUACAGAGCUATT |
| shDNMT2-1 | GCGCTGAGAGAAAGCTGTATA |
| shDNMT2-2 | GCCAAGACGATTGAAGGCATT |
| shDNMT2-3 | CCAAAGTCATTGCTGCGATAT |
| mimics-5’tiRNA^Gly-GCC^ | GCAUAGGUGGUUCAGUGGUAGAAUUCUUGCC |
| inhibitor-5’tiRNA^Gly-GCC^ | GGCAAGAAUUCUACCACUGAACCACCUAUGC |
