# Supplementary material for: DNMT2 inhibits anaplastic thyroid cancer progression by downregulating 5’tiRNAGly-GCC production
Source: Cell Death Dis. 2026 Feb 21;17(1):240. doi: 10.1038/s41419-026-08488-5 (PMC12949022; doi:10.1038/s41419-026-08488-5)
Supplement: Supplementary file 3 — Supplemental Table 2. Primer sequences [file 41419_2026_8488_MOESM3_ESM.docx]

Supplemental Table 2. Primer sequences

| NCBI gene symbol/Name | Forward (5'→3') | Reverse (3'→5') | RT Primer |
| --- | --- | --- | --- |
| GAPDH | GGAGCGAGATCCCTCCAAAAT | GGCTGTTGTCATACTTCTCATGG | / |
| U6 | CTCGCTTCGGCAGCACA | AACGCTTCACGAATTTGCGT | / |
| DNMT2 | GATGTCAACACTGTCGCTAATGA | CCTTCAATCGTCTTGGCAAGT | / |
| hnRNPH1 | ATTCAAAATGGGGCTCAAGGTAT | GTGTCAGGACTATTTGGACCAG | / |
| ANG | CAAGGCCATCTGTGAAAACAAG | CAGGGGGAACCTCCATGTAG | / |
| tRNA-Gly-GCC | AGGAGGCCCAGGTTTGATTCC | AGTGCAGGGTCCGAGGTATT | GTCGTATCCAGTGCAGGGTCCGAGGTATTCGCACTGGATACGACGGCAAG |
| 5’tiRNA^Gly-GCC^ | GCATAGGTGGTTCAGTGGTAGAATT | AGTGCAGGGTCCGAGGTATT | GTCGTATCCAGTGCAGGGTCCGAGGTATTCGCACTGGATACGACTGGTGC |
