# Supplementary material for: DNMT2 inhibits anaplastic thyroid cancer progression by downregulating 5’tiRNAGly-GCC production
Source: Cell Death Dis. 2026 Feb 21;17(1):240. doi: 10.1038/s41419-026-08488-5 (PMC12949022; doi:10.1038/s41419-026-08488-5)
Supplement: Supplementary file 4 — Supplementary material-Original Blots [file 41419_2026_8488_MOESM4_ESM.pdf]

DNMT2 inhibits anaplastic thyroid cancer progression by downregulating 5'tiRNA$^{Gly-GCC}$ production

Supplemental Material – Original Western Blotting Images

Relevant areas for cropped blots in the main and extended Data figures are shown with red boxes.

Supplemental Material to Fig. 1 (original western blotting images)

Figure 1F

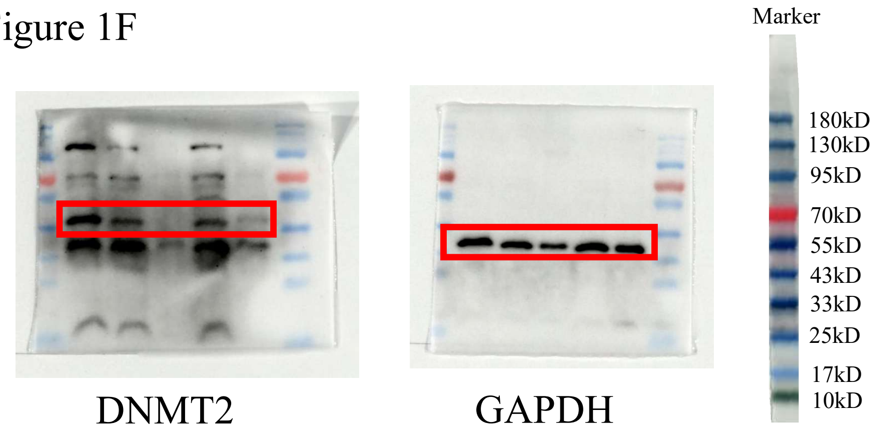

Figure 1G

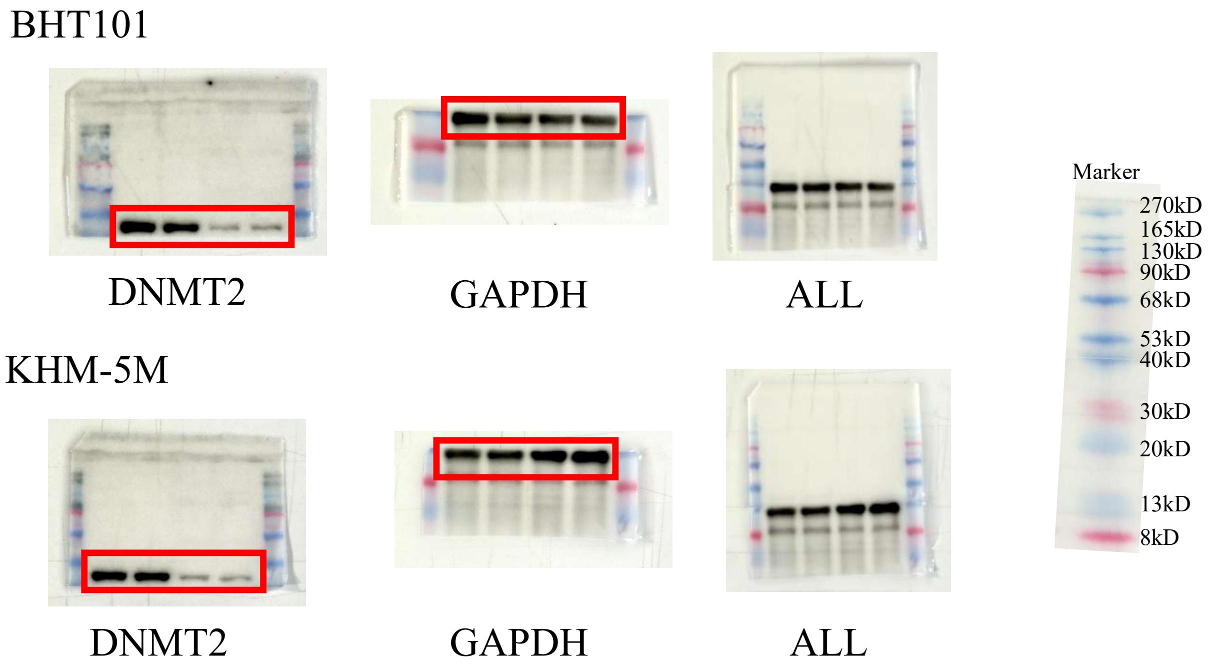

Figure 1H

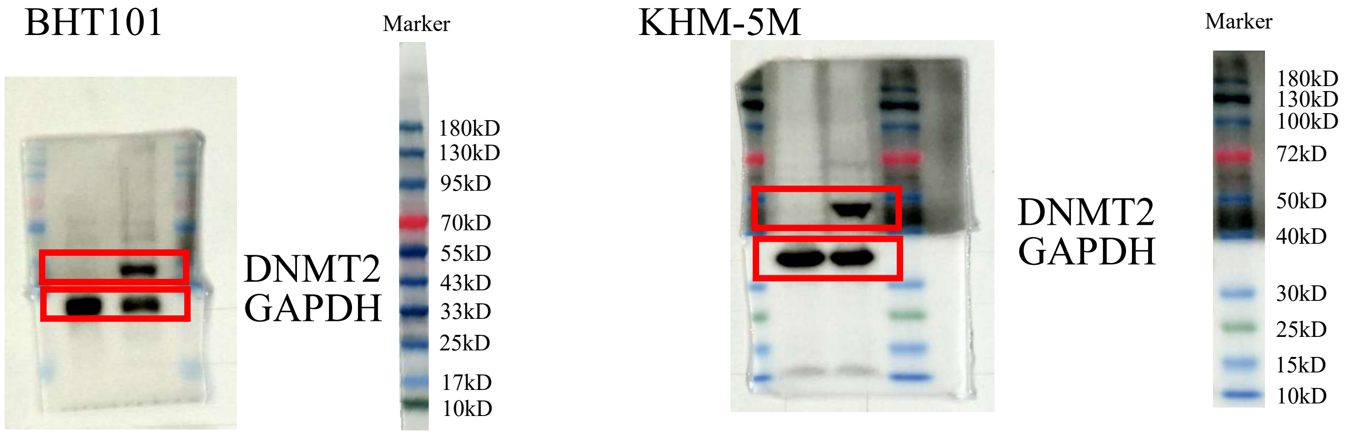

Supplemental Material to Fig. 2 (original western blotting images)

Figure 2E

BHT101

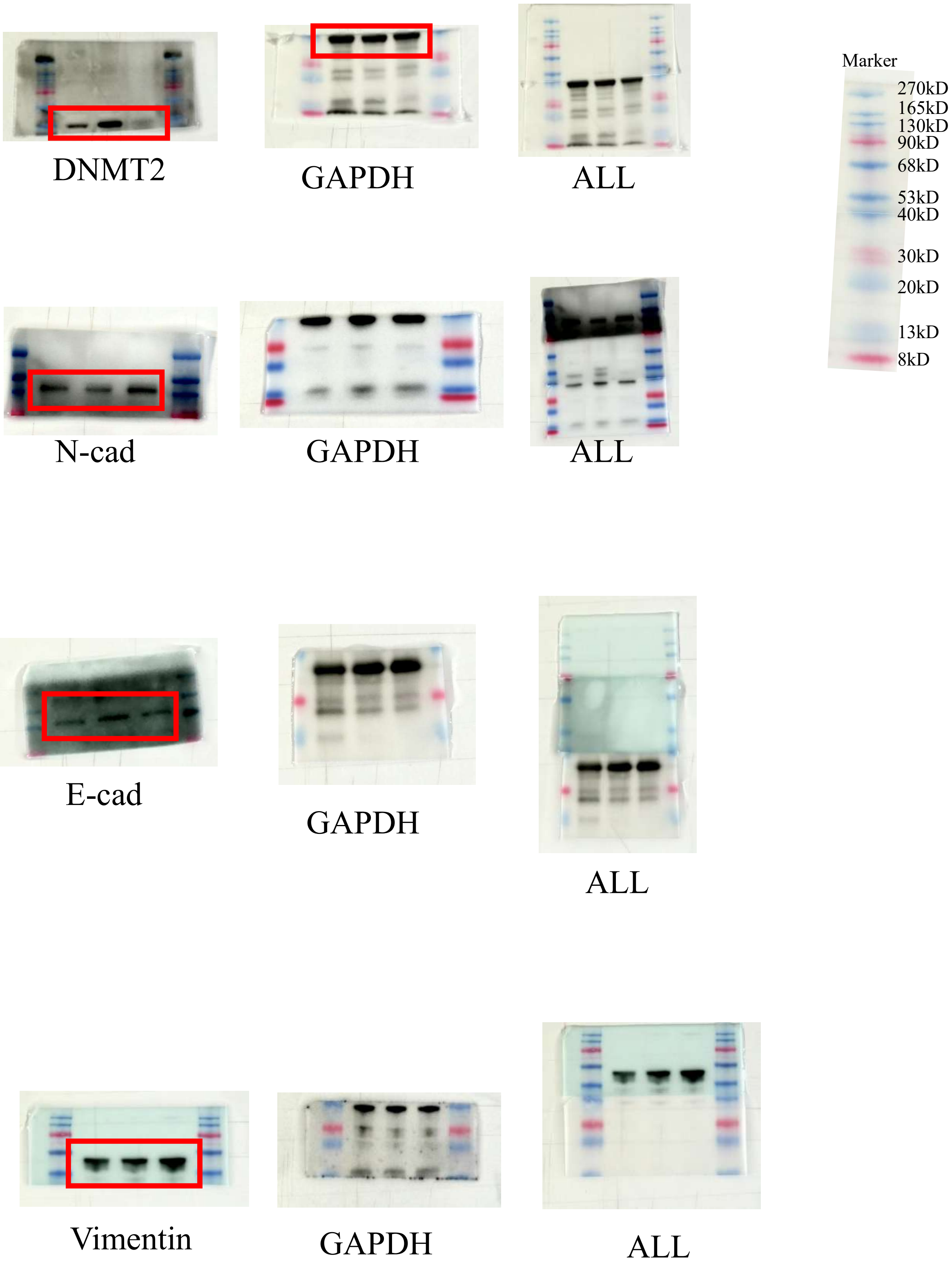

Supplemental Material to Fig. 2 (original western blotting images)

Figure 2E

KHM-5M

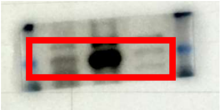

DNMT2

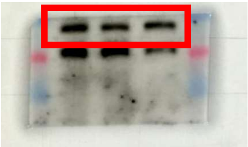

GAPDH

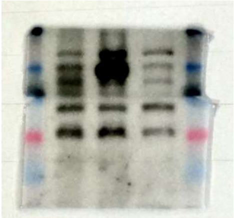

ALL

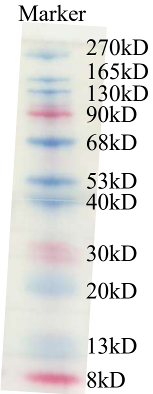

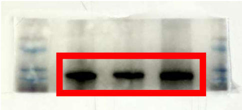

N-cad

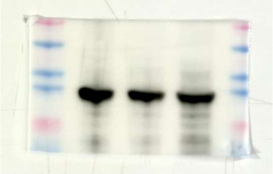

GAPDH

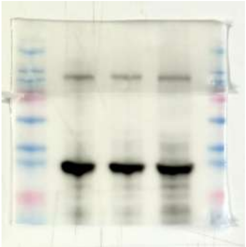

ALL

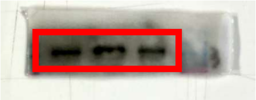

E-cad

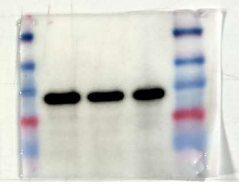

GAPDH

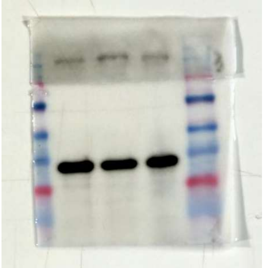

ALL

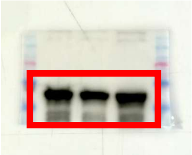

Vimentin

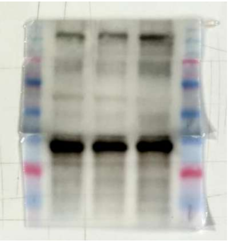

ALL

Supplemental Material to Fig. 2 (original western blotting images)

Figure 2G

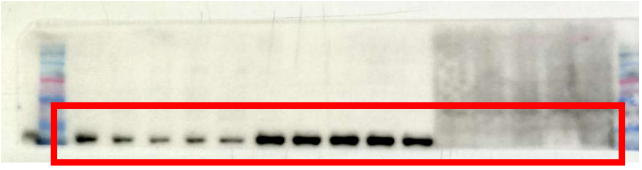

DNMT2

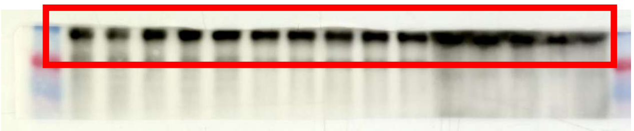

GAPDH

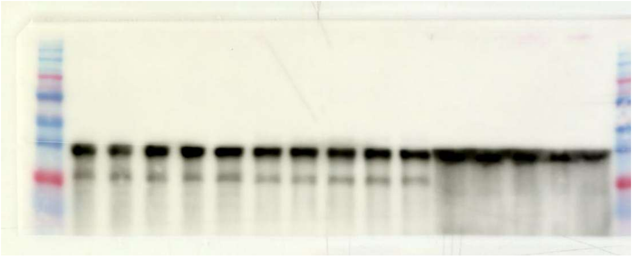

ALL

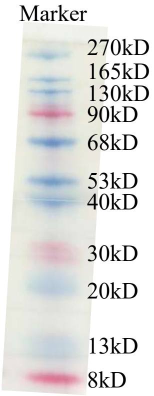

Supplemental Material to Fig. 5 (original western blotting images)

Figure 5F

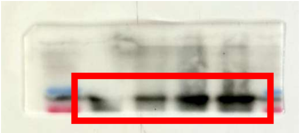

GAPDH

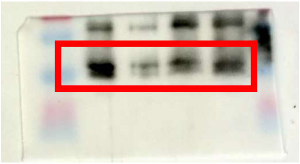

ANG

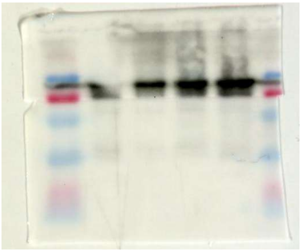

ALL

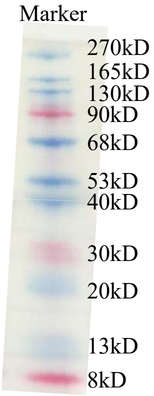

Figure 5G

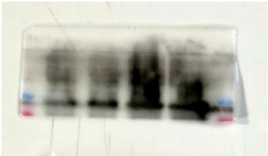

GAPDH

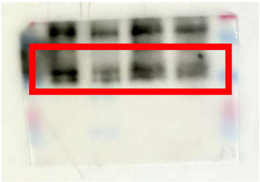

ANG

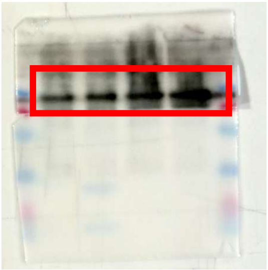

ALL

Figure 5H

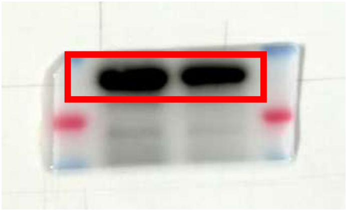

GAPDH

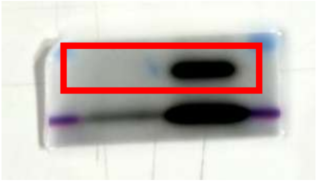

ANG

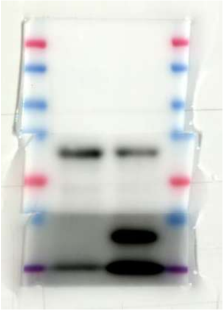

ALL

Figure 5I

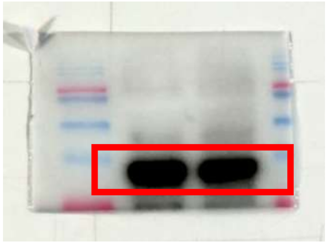

GAPDH

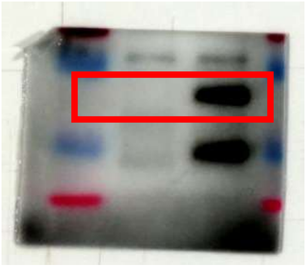

ANG

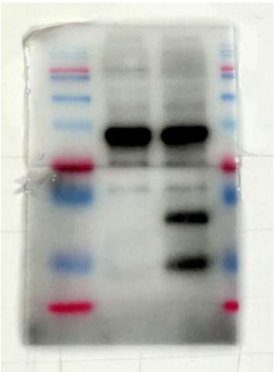

ALL

Supplemental Material to Fig. 7 (original western blotting images)

Figure 7L

BHT101

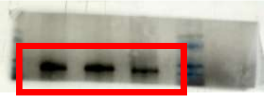

N-cad

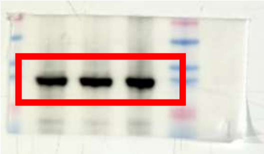

GAPDH

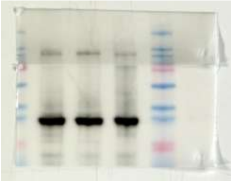

ALL

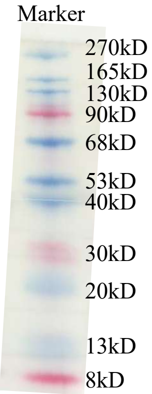

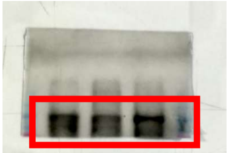

E-cad

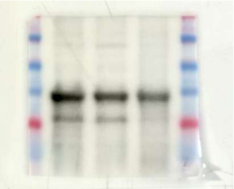

GAPDH

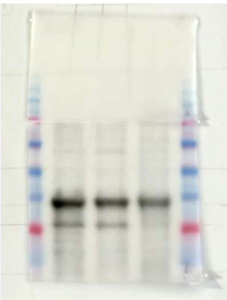

ALL

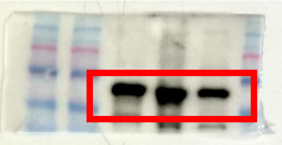

Vimentin

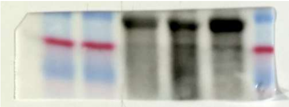

GAPDH

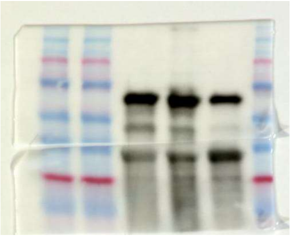

ALL

Supplemental Material to Fig. 6 (original western blotting images)

Figure 7L

KHM-5M

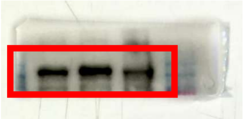

N-cad

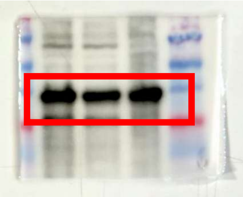

GAPDH

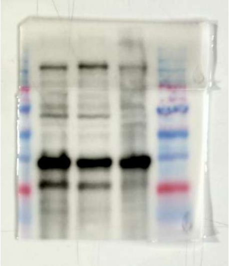

ALL

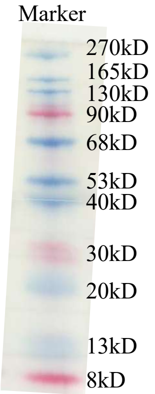

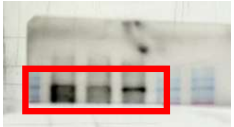

E-cad

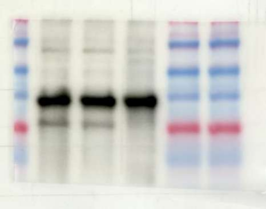

GAPDH

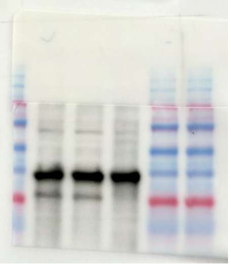

ALL

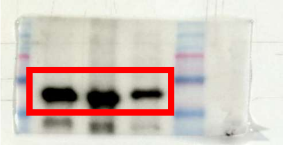

Vimentin

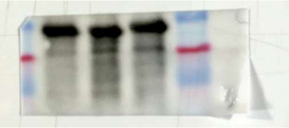

GAPDH

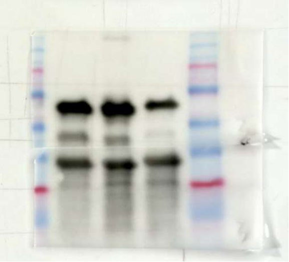

ALL

Supplemental Material to Fig. 8 (original western blotting images)

Figure 8F

BHT101

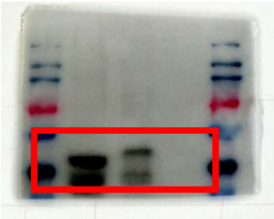

hnRNPH1

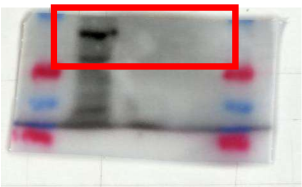

GAPDH

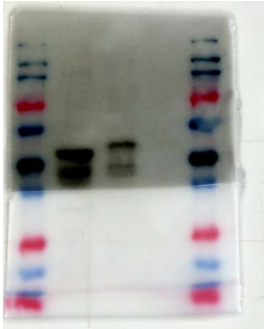

ALL

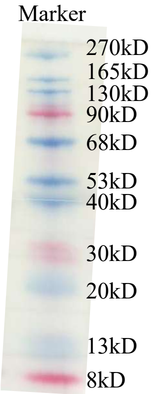

KHM-5M

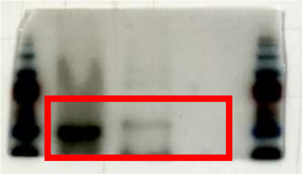

hnRNPH1

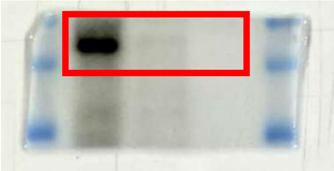

GAPDH

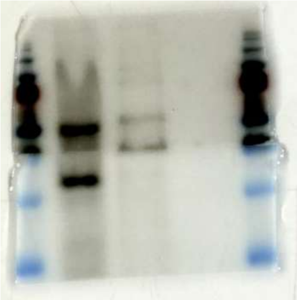

ALL

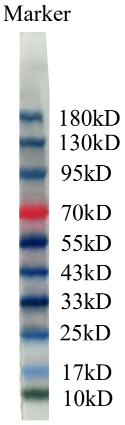

Figure 8K

BHT101

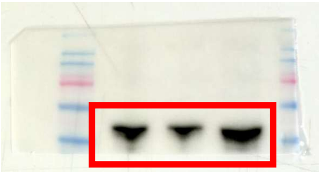

hnRNPH1

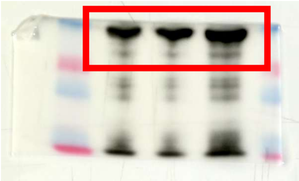

GAPDH

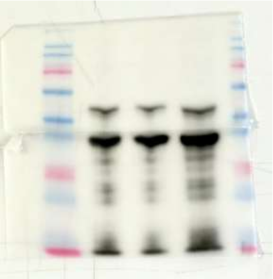

ALL

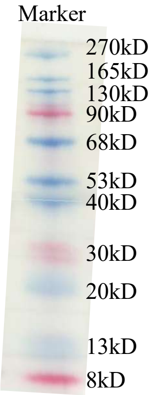

KHM-5M

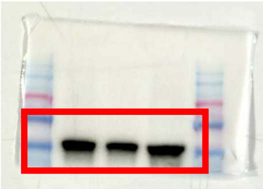

hnRNPH1

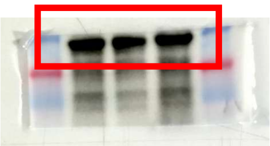

GAPDH

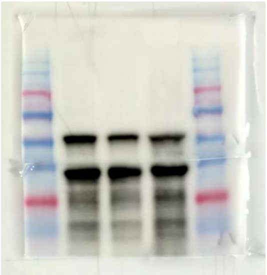

ALL

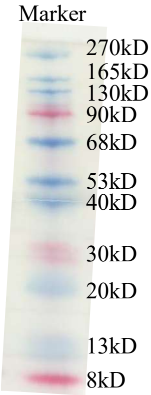

Supplemental Material to Fig. 8 (original western blotting images)

Figure 8L

BHT101

LAMIN B1

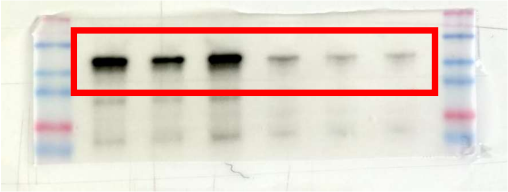

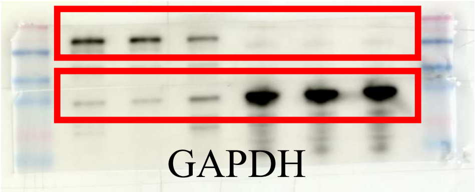

Marker

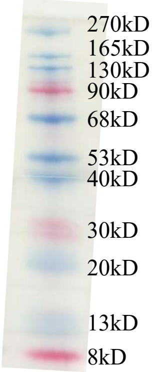

hnRNPH1

KHM-5M

LAMIN B1

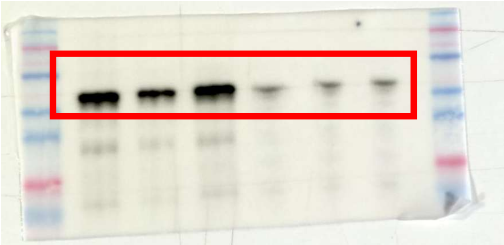

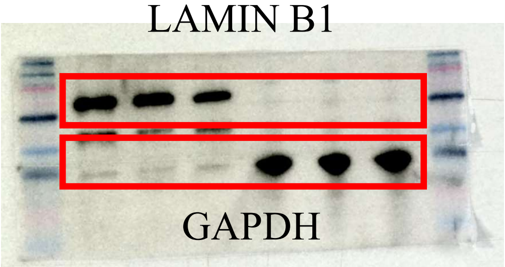

hnRNPH1

Supplemental Material to Supplemental Figure 5 (original western blotting images)

Supplemental Figure 5D

BHT101

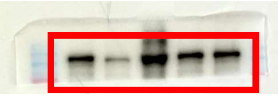

N-cad

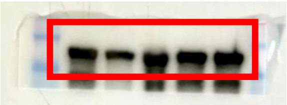

Vimentin

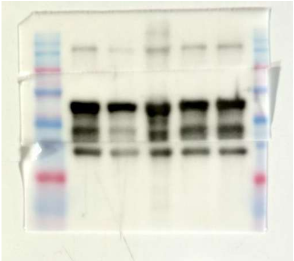

ALL

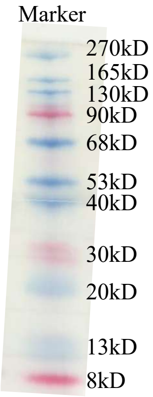

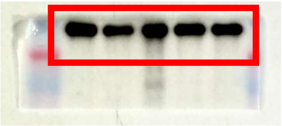

GAPDH

KHM-5M

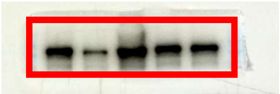

N-cad

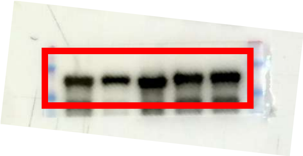

Vimentin

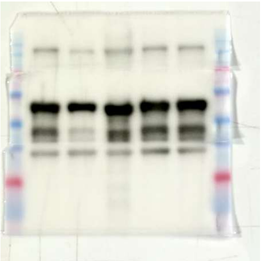

ALL

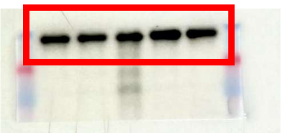

GAPDH

Supplemental Material to Supplemental Figure 7 (original western blotting images)

Supplemental Figure 7C

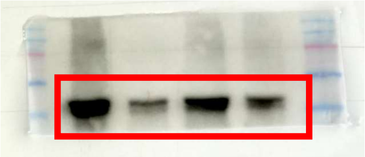

hnRNPH1

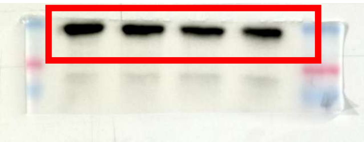

GAPDH

Marker

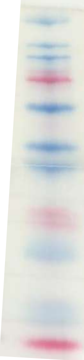

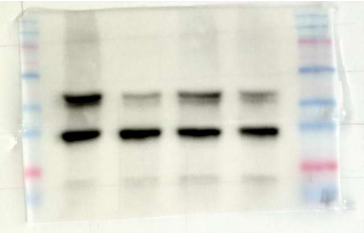

ALL

Supplemental Figure 7D

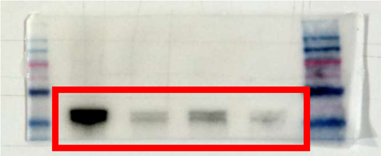

hnRNPH1

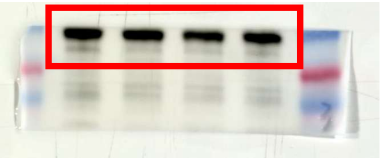

GAPDH

Marker

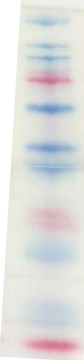

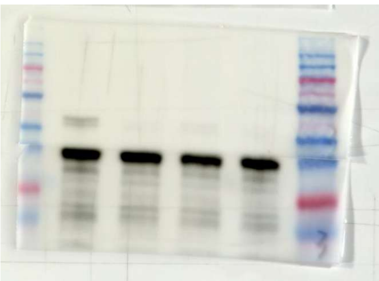

ALL

Supplemental Figure 7E

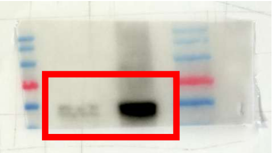

hnRNPH1

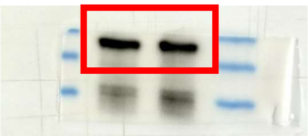

GAPDH

Marker

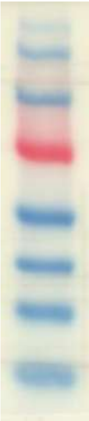

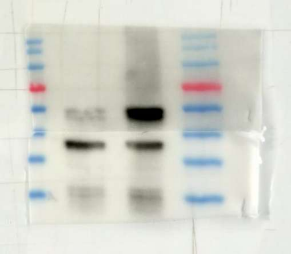

ALL

Supplemental Material to Supplemental Figure 7 (original western blotting images)

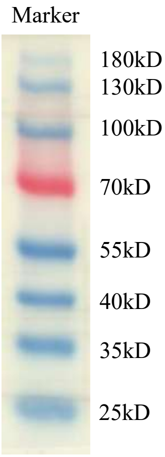

Supplemental Figure 7F

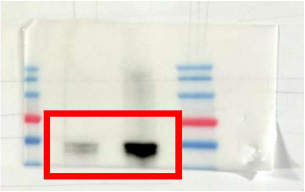

hnRNPH1

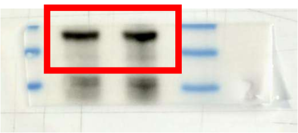

GAPDH

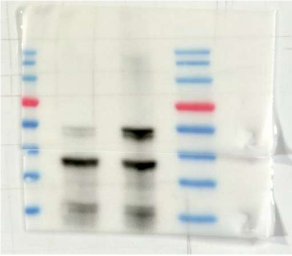

ALL

Supplemental Figure 7G

BHT101

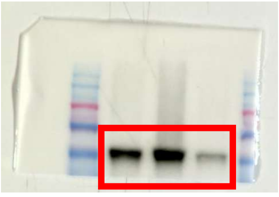

hnRNPH1

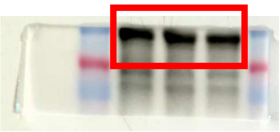

GAPDH

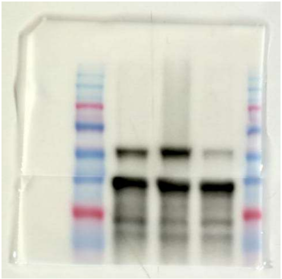

ALL

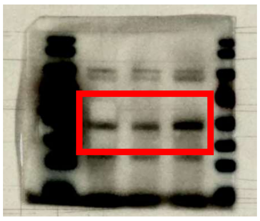

PD-L1

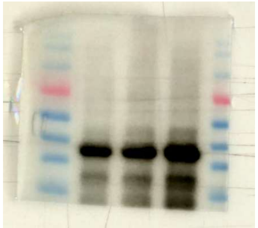

GAPDH

KHM-5M

Supplemental Material to Supplemental Figure 7 (original western blotting images)

Supplemental Figure 7G

KHM-5M

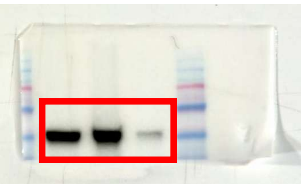

hnRNPH1

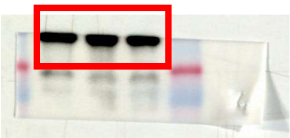

GAPDH

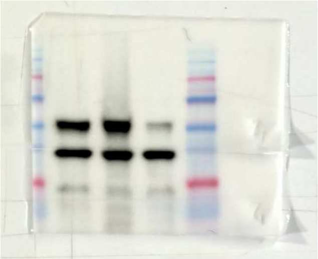

ALL

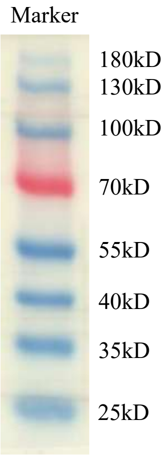

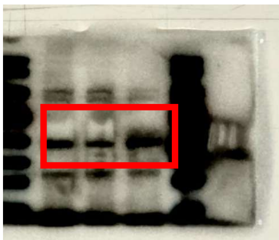

PD-L1

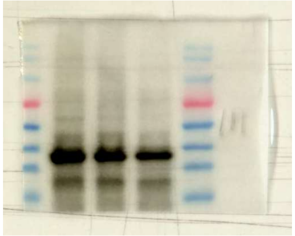

GAPDH

Supplemental Figure 7H

BHT101

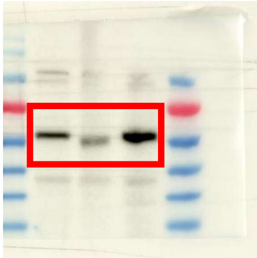

PD-L1

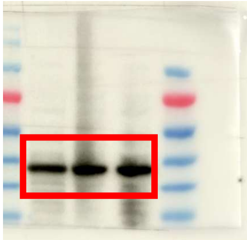

GAPDH

KHM-5M

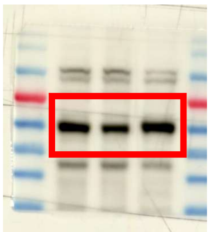

PD-L1

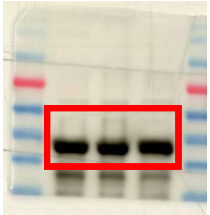

GAPDH

Supplemental Material to Supplemental Figure 7 (original western blotting images)

Supplemental Figure 7I

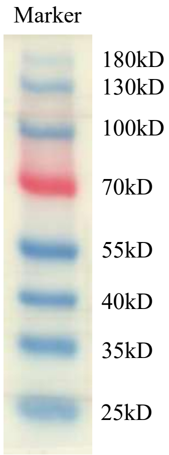

BHT101

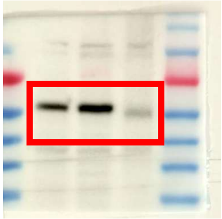

PD-L1

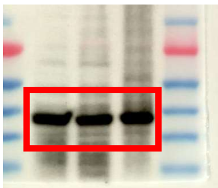

GAPDH

KHM-5M

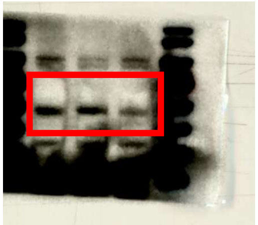

PD-L1

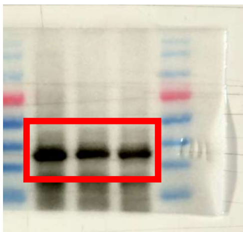

GAPDH

Supplemental Figure 7J

BHT101

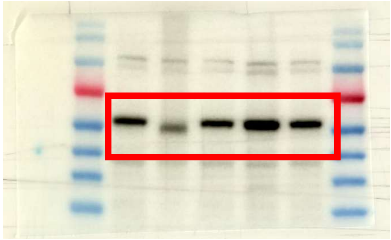

PD-L1

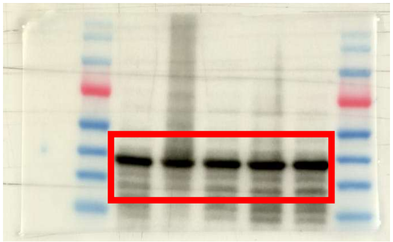

GAPDH

KHM-5M

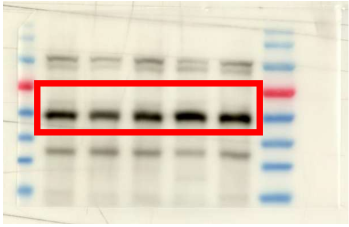

PD-L1

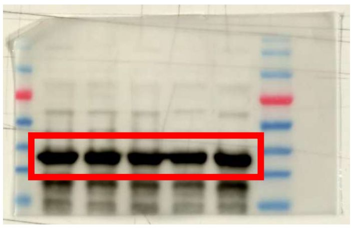

GAPDH
